# On-demand EEG education through competition – A novel, app-based approach to learning to identify interictal epileptiform discharges

**DOI:** 10.1016/j.cnp.2023.08.003

**Published:** 2023-08-19

**Authors:** Jaden D. Barfuss, Fábio A. Nascimento, Erik Duhaime, Srishti Kapur, Ioannis Karakis, Marcus Ng, Aline Herlopian, Alice Lam, Douglas Maus, Jonathan J. Halford, Sydney Cash, M. Brandon Westover, Jin Jing

**Affiliations:** a Department of Neurology, Massachusetts General Hospital, Harvard Medical School, Boston, MA, USA; b Centaur Labs, Boston, MA, USA; c Department of Neurology, Emory University School of Medicine, Atlanta, GA, USA; d Section of Neurology, Department of Internal Medicine, Health Sciences Centre, University of Manitoba, Winnipeg, MB, Canada; e Division of Epilepsy, Department of Neurology, Yale University, New Haven, CT, USA; f Medical University of South Carolina, Charlestown, SC, USA

**Keywords:** EEG, Epilepsy, Education, Interictal epileptiform discharges

## Abstract

**Objective:**

Misinterpretation of EEGs harms patients, yet few resources exist to help trainees practice interpreting EEGs. We therefore sought to evaluate a novel educational tool to teach trainees how to identify interictal epileptiform discharges (IEDs) on EEG.

**Methods:**

We created a public EEG test within the iOS app DiagnosUs using a pool of 13,262 candidate IEDs. Users were shown a candidate IED on EEG and asked to rate it as epileptiform (IED) or not (non-IED). They were given immediate feedback based on a gold standard. Learning was analyzed using a parametric model. We additionally analyzed IED features that best correlated with expert ratings.

**Results:**

Our analysis included 901 participants. Users achieved a mean improvement of 13% over 1,000 questions and an ending accuracy of 81%. Users and experts appeared to rely on a similar set of IED morphologic features when analyzing candidate IEDs. We additionally identified particular types of candidate EEGs that remained challenging for most users even after substantial practice.

**Conclusions:**

Users improved in their ability to properly classify candidate IEDs through repeated exposure and immediate feedback.

**Significance:**

This app-based learning activity has great potential to be an effective supplemental tool to teach neurology trainees how to accurately identify IEDs on EEG.

## Highlights

• An immediate feedback method for learning to identify IEDs has great potential to be an effective supplemental EEG educational tool.
• On average users improved their accuracy in identifying IEDs on EEG by 13% over 1000 questions and had an ending accuracy of 81%.
• 10% of users reached an accuracy level at or above the average accuracy (90%) of 9 experts who participated in a similar test.

## 1 Introduction

A large portion of EEG studies in the U.S. are read by general neurologists without post-residency/fellowship training in neurophysiology or epilepsy. Given the well-known challenges of distinguishing benign from pathological EEG features, coupled with worldwide deficiencies in neurology residency EEG education (Lourenço et al., 2021, Nascimento and Gavvala, 2021), EEG misinterpretation is not uncommon in real-world practice. Inaccurate EEG reads may result in serious consequences to patients, especially in scenarios where a diagnosis of epilepsy and initiation of an antiseizure medication rely on EEG results (Krumholz et al., 2015). Consequently, misinterpretation of EEG due to under-calling (failure to recognize IEDs when present) or over-calling (mistakenly reporting benign transients as IEDs) can lead to misdiagnosis and harm to patients. Improving EEG education is thus a necessary step to improving epilepsy patient care.

In this study, we sought to (i) investigate learnability of epileptiform discharge identification through repeated exposure to reading potential IEDs with immediate feedback from a smartphone-based application in the form of a competition and (ii) compare features of candidate IEDs involved in decision-making used by experts and non-experts in the classification of candidate IEDs. Our goal was to create a novel EEG teaching tool that is educational, easy to use, and entertaining. This EEG tool was used by a total of 2,270 participants, most of whom increased their accuracy significantly over a relatively short period of time, many reaching near-expert levels of performance.

## 2 Methods

### 2.1 Study design

*Gold standard.* In previous work, we collected 13,262 candidate IEDs and recruited eight physician experts to annotate each candidate (Jing et al., 2020). We refer readers to this reference for a detailed discussion on how the 13,262 candidate IEDs were collected. Each expert had at least 1 year of fellowship training in clinical neurophysiology (experience in reading EEGs of 4–16 years, median of 9.5 years). Experts independently reviewed each candidate IED in a customized graphical user interface which allowed experts to change EEG montage, gain, and filters, and were asked to classify each candidate IED as epileptiform (an IED) or not (Jing et al., 2020). For the present study, we considered a candidate IED “positive” if at least 3 of the 8 original experts classified it as an IED. Nevertheless, we emphasize that this cutoff is somewhat arbitrary. In acknowledgement of this, we also evaluated performance on various categories of candidate IEDs, based on the degree of agreement among the original 8 experts: clear IEDs (6–8 of 8 votes), clear non-IEDs (0–2 of 8 votes), or unclear IEDs (3–5 of 8). We used the 13,262 IED candidates and expert votes as the gold standard for this public competition.

**Fig. 1:**
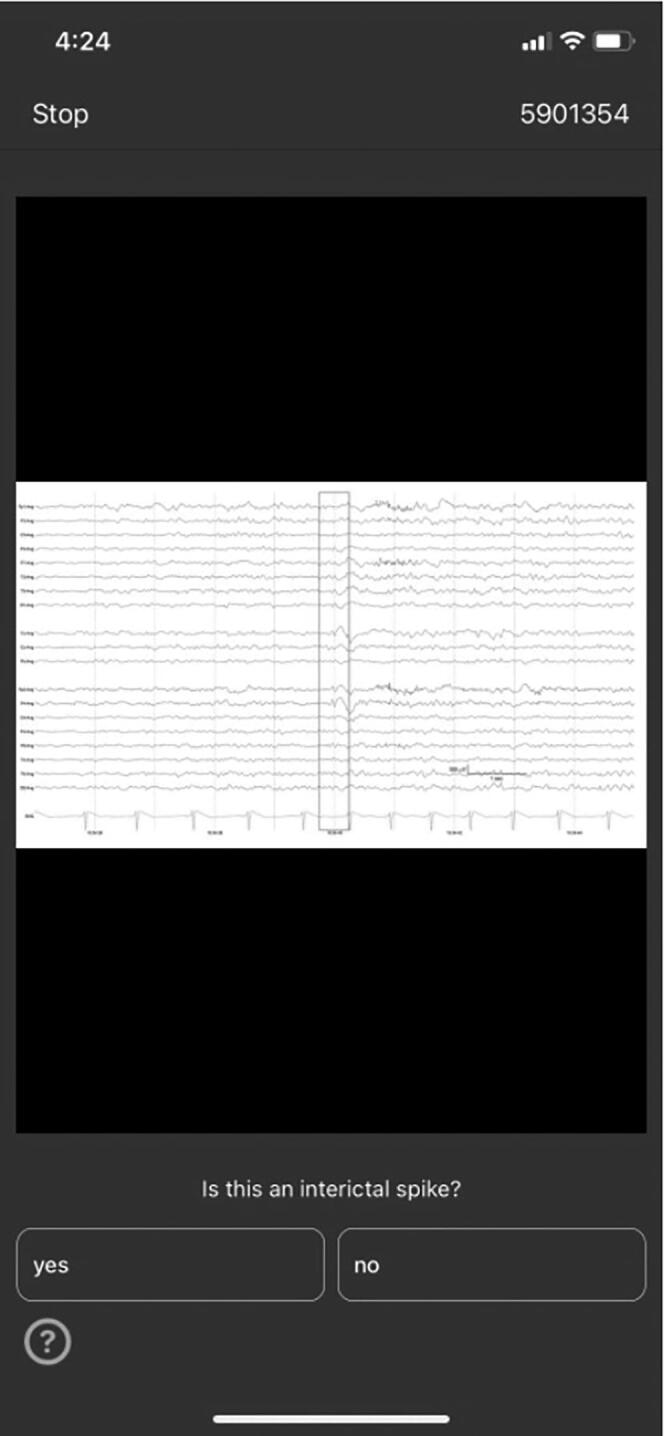
Application-based EEG competition. Legend. Representative screenshot of EEG competition on DiagnosUs app.

*IED Scoring Contest.* Participants/users were shown randomly selected images from our 13,262 candidate IEDs. Each image showed a 10-second EEG epoch with a vertical red rectangle highlighting the candidate IED (Fig. 1). Epochs were shown as static images, in bipolar montage. Users were asked to vote yes (IED) or no (non-IED) for as many candidate waveforms as they were willing to evaluate. After rating each candidate IED, users were given instant feedback based on the expert consensus gold standard. Prior to starting to play, users were required to complete a short training with a tutorial on epileptiform discharges and 25 practice questions. Notably, answers to the practice questions were not included in users’ performance. During the competition, time-limited contests were intermittently launched to maintain user interest. There were two types of contests. The first awarded users with best accuracies, whereas the second rewarded users who correctly answered the maximum number of consecutive questions. Both contests included a publicly visible leaderboard (in the app), and prizes ranged from $0.5 to $75. The competition was free of charge and open to anyone who had access to an iOS-compatible device (iPhone/iPad) to run the application.

*Contest Participants.* We instituted an iOS app (DiagnosUs, https://www.DiagnosUs.com) competition in collaboration with Centaur Labs (Boston, MA, www.centaurlabs.com). The competition was available to all users of the app including physicians, advanced practice providers, medical students, and others.

### 2.2 Standard protocol approvals, registrations, and patient consents

Preparation of the data and public sharing of the deidentified images of candidate IEDs on the app was conducted under an IRB approved protocol. The study data was deidentified and obtained from users who volunteered to participate in a public EEG test within the iOS app DiagnoUs. Use of the app did not require users to provide written informed consent.

### 2.3 Statistical analysis

Our statistical analysis had three aims: (1) learning rate analysis: characterize the rate of improvement in users’ ability to accurately classify candidate IEDs; (2) feature analysis: determine which EEG waveform features correlate with user voting behavior; and (3) expert vs crowd disagreement analysis: qualitatively analyze candidate IED features in cases where competition participants strongly disagreed with the gold standard. The latter aimed to gain insights into how we might design future contests to optimize learning for difficult cases.

*Learning rate analysis.* We estimated the learning rate of each user by fitting their binary response (voting) data (correct = 1, incorrect = 0) using the following equation:

$$p\left(n\right)=a{\left(\frac{n}{N}\right)}^{b}$$

In this model, p(n) is the probability of a correct response as a function of the number n of questions answered; N represents a “large” number at which learning is assumed to saturate (we set N = 5,000; the large majority of participants completed <1,000 questions); b is the learning rate (on a logarithmic scale: log(p) ∼ b ⋅ log(n) + const); $a/N^{b}$ is the participants’ initial accuracy; and a is the asymptotic accuracy (after answering N questions). This simple model empirically provides a good fit to most participants’ sequential binary response data, and describes a learning process in which learning initially proceeds rapidly then continues much more slowly. We limited analysis to users who answered at least 100 questions, which appeared to satisfactorily fit the learning model in most cases. The model’s two parameters a and b for each participant were found by maximum likelihood estimation, i.e., by maximizing $L\left(a,b\right)=\sum_{n=1}^{N}\log p\left(n\right)$.

We used this model to analyze how accuracy, false and true positive rates evolved with practice. We limited analysis of false positive rates to “clear” non-IEDs (cases with 0–2 of 8 votes in the gold standard); and analysis of sensitivity to clear IEDs (cases with 6–8 of 8 votes in the gold standard). We grouped users into quantiles (75–95%, 50–75%, 25–50%, 5–25%, 0–5%) based on their model-projected accuracy after 1,000 questions to visually compare performance for clear non-IEDs, clear IEDs, overall, and with reference to expert levels of performance. Additionally, users were stratified by profession (neurologist, neurologist-epileptologist, or non-neurologist/non-epileptologist) and whether they reported reading EEGs regularly.

As a benchmark we calculated accuracies and true and false positive rates for both the original 8 “gold standard” experts, and for 9 experts who were not part of the gold standard (the “additional 9”, A9; experience in reading EEGs of 0.6 to 35 years, median of 6 years). These 9 experts were physicians/neurologists who were either undergoing or had undergone clinical neurophysiology fellowship training. We used an approach similar to the one used by participants in the present study, except that the A9 experts evaluated spikes in a web application rather than on a phone or tablet and were able to switch montages and adjust the gain and sensitivity of the EEG. We resampled questions answered by the A9 experts to match the proportions of clear IEDs, clear non-IEDs, and unclear IEDs seen by participants in the current study. The mean accuracy curve of the A9 started at 81% and ended at 90% accuracy. This was used to establish the expert level for our study: 90%. Notably, data from all 9 experts (A9) were used to determine the expert level even though these experts’ experience in EEG reading varied widely. This is justified because our estimate of expert level performance did not change significantly (range of 90 to 92.5%) in sensitivity analysis where we calculated multiple expert levels by varying cutoffs of experience in EEG reading to be considered an expert (Supplemental Fig. 2). We excluded the original 8 experts from the definition of the expert level because their responses were used to create the gold standard.

To explore how the ‘wisdom of the crowd’ compared with experts, we pooled votes across participants to generate a crowdsourced label for each candidate IED. To generate the crowd’s vote, we weighted each participant’s vote by their overall accuracy on all questions answered, and added up all weighted votes to obtain a score for each option (IED or non-IED). The crowdsourced vote for that sample was then assigned as the option with the highest score.

**Fig. 2:**
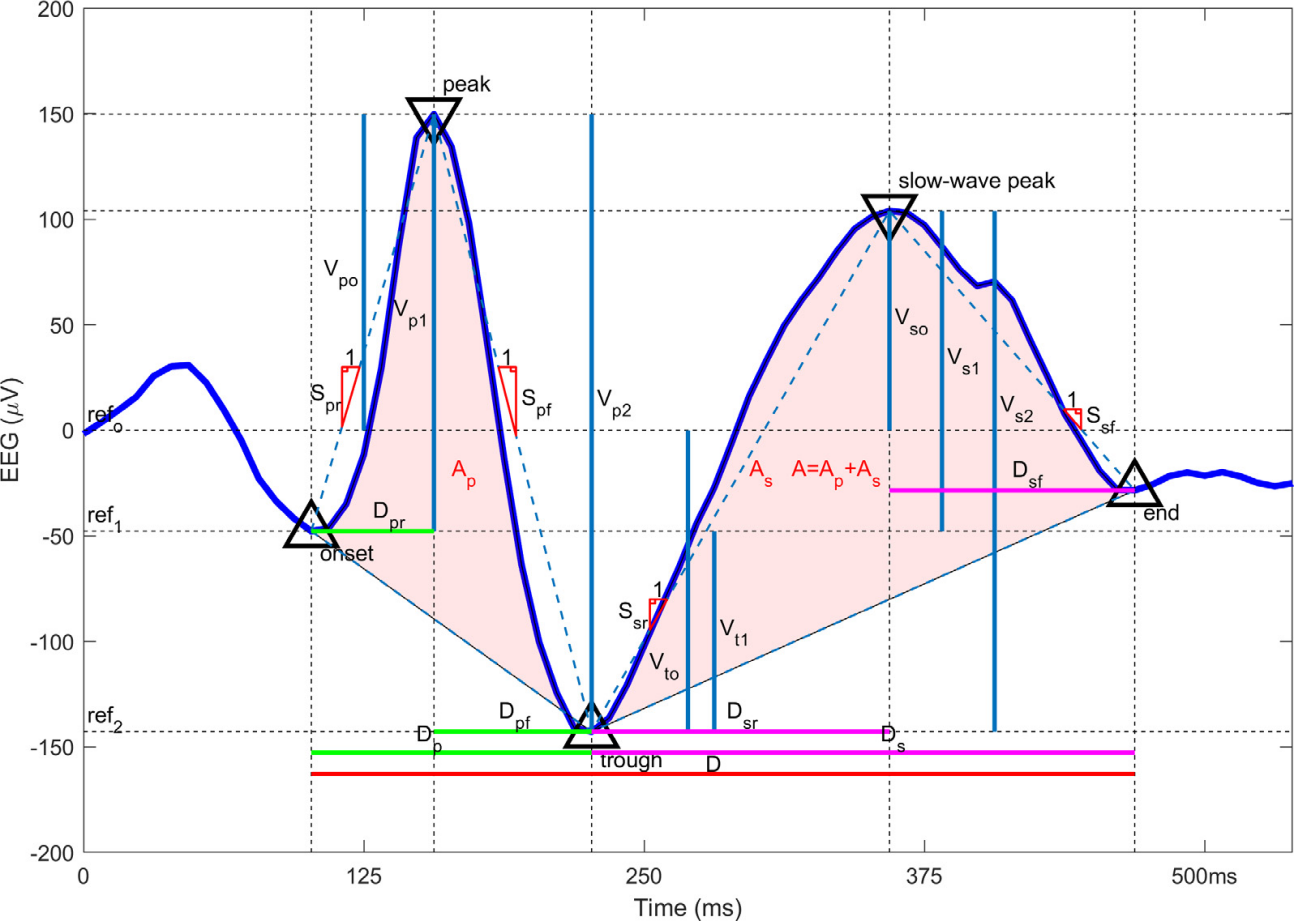
Twenty-three morphological features of interictal epileptiform discharges. Legend. V_p0_ = voltage at peak, V_p1_ = difference in voltage between peak and onset, V_p2_ = difference in voltage between peak and trough, V_t0_ = voltage at trough, V_t1_ = difference in voltage between trough and onset, V_s0_ = voltage at slow wave peak, V_s1_ = difference between slow wave peak and onset, V_s2_ = difference in voltage between slow wave peak and trough, S_pr_ = rising slope of peak, S_pf_ = falling slope of peak, S_sr_ = rising slope of slow wave, S_sf_ = falling slope of slow wave, D = duration of IED, D_p_ = duration of peak, D_pr_ = duration of rising half of the peak, D_pf_ = duration of the falling half of the peak, D_s_ = duration of the slow wave, D_sr_ = duration of the rising half of the slow wave, D_sf_ = duration of the falling half of the slow wave, A = area under the IED, A_p_ = Area under the peak, A_s_ = area under the slow wave, MaxCorr = correlation between EEG channels (not shown in figure).

*Feature analysis****.*** We investigated which features of IED morphology correlated with participant voting behavior. For this analysis we considered a broad set of 23 waveform features used to characterize IEDs, described in prior literature (Jing et al., 2020). These features are divided into 5 general categories including (1) voltage amplitudes, (2) durations, (3) slopes, (4) areas, and (5) across-channel correlation (Fig. 2).

To understand which features users implicitly relied on to decide whether a waveform was epileptiform, we ranked features according to how strongly their values correlated with participant agreement about whether candidate IEDs were epileptiform. For this analysis we used user responses after the 200th evaluated candidate IED because, empirically, this appeared to be a point beyond which most users had substantially improved beyond their initial starting point (thus likely that by this point most participants had stabilized in their learning of IED features). We created 9 bins for candidate IEDs based on the percent of users who classified them as IEDs: (0–11.2%, 11.2–22.3%, … 88.9–100%); these bins were chosen to roughly correspond to the 9 different possible levels of agreement among the original 8 experts (0/8, 1/8, …, 8/8). We calculated a correlation coefficient for each of the 23 features using the median feature value of the candidate IEDs in each of the 9 bins. We then compared the correlation between feature values and agreement among participants with the same correlation between feature values and agreement among the original 8 experts.

*Expert* vs*. crowd disagreement analysis****.*** Three of the authors (BW, JJ, FN; experience in reading EEG of 1 to 10 years) performed a qualitative visual analysis of the candidate IEDs in which the majority of participants (“the crowd”) disagreed with experts (hereon referred to as the “outliers”) in order to understand the causes underlying this disagreement. Major disagreement was defined when a potential IED fit two requirements. First, in situations where (i) experts rated candidate IEDs as IED (4–8/8 votes) while most users did not do so (less than the minimum line on the box and whisker plot: 1.5 time the interquartile range below the 25th percentile), or (ii) experts rated candidate IEDs as non-IED (0–2/8 votes) while most users rated them as IED (more than the maximum line on the box and whisker plot: 1.5 times the interquartile range above the 75th percentile). Second, in order to limit the otherwise large amount of outliers of IEDs with 7–8/8 expert votes, in situations where experts rated candidate IEDs as IED (7–8/8 votes) while <50% of users did so. During this qualitative analysis, we visualized the outliers in a graphical user interface with the ability to change between montages but no ability to adjust filters or gain to appreciate subtle features that might not have been apparent in the static images used in the app-based learning tool. We evaluated each of the outliers to qualitatively identify features that might explain why these examples were difficult for the crowd to classify correctly. Based on this qualitative analysis we categorized all outliers into 2 groups and 6 subgroups. We describe the features of each subgroup below.

## 3 Results

A total of 2,270 people participated in our EEG teaching tool. They collectively answered 1,101,811 questions between July 2019 and April 2021. A single user answered enough questions in the 13,262-EEG dataset to have seen the same candidate IED more than once. Nine hundred and one (40%) participants answered at least 100 questions and were included in the analysis. The remaining participants were excluded. Of the 901 participants, 136 (15%) were healthcare professionals, 384 (43%) were healthcare students, 322 (36%) were in professions outside of healthcare, and the remaining 59 (7%) did not disclose their backgrounds (full breakdown Supplemental Table 1). Further, 118 (13%) of all participants reported reading EEG regularly while 742 (82%) reported not doing so.

**Fig. 3:**
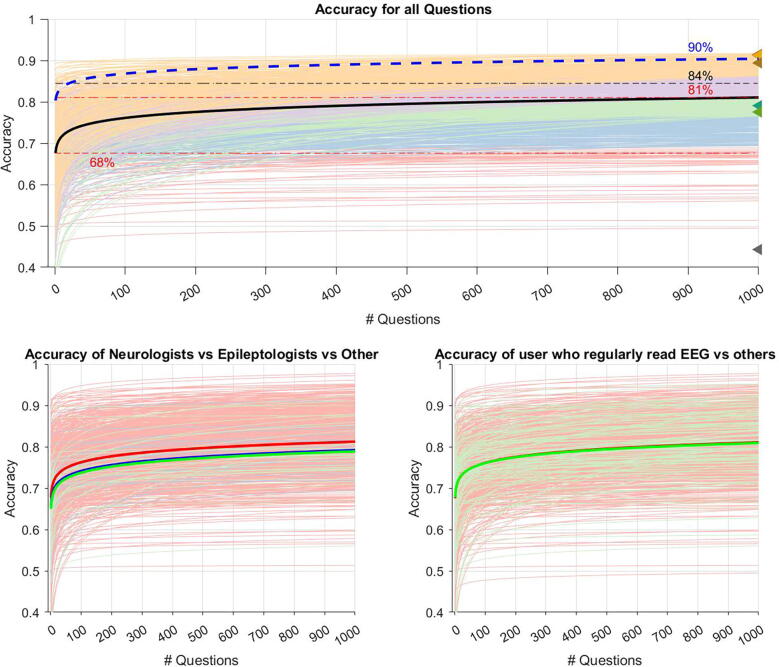
Users’ overall learning curves. Legend. Predicted learning curves representing the accuracy improvement for each user as estimated by the equation a(n/N)^b^ (a = accuracy at question 5000, N = 5000, b = learning rate, n = question number). (A) User’s learning curves were grouped into 5 quantiles based on predicted accuracy at 1000 questions. Orange (75% − 95%), purple (50% − 75%), green (25% − 50%), blue (5% − 25%), red (0% − 5%). The black line shows the mean accuracy value at each question number. The predicted mean accuracy of users at the start and at 1000 questions is shown by the red dashed line at 68% and 81%. The accuracies of the 8 experts predicted by SpikeNet are each graphed by a triangle marker on the right side of the plot (44%, 78%, 79%, 89%, 91%, 91%, 91%, 91%). The results of the additional 9 experts are shown by the blue dashed line starting at 81% and ending at 90%. The results of the crowd sourcing are shown by the black dashed line at 84%. (B) Users were grouped by profession. non-epileptologists neurologists (blue), epileptologists (green), and non-neurologist/non-epileptologist (red). (C) Users were grouped by whether they regularly read EEGs (green) or not (red). (For interpretation of the references to color in this figure legend, the reader is referred to the web version of this article.)

Learning rate analysis of responses from the 901 analyzed participants showed that, on average and in the course of answering 1,000 questions, participants improved by 13% (95% CI [0.13, 0.14], p < 0.001, **Supplemental** Table 2) and achieved a mean ending accuracy of 81%. By comparison, the A9 showed a mean improvement of 9% and a mean ending accuracy of 90%. Based on crowdsourcing, we found a weighted accuracy of the crowd to be 84% (Fig. 3**A**).

Subgroup analysis showed that participants who were non-neurologists/non-epileptologists (91%) improved by 14% with a mean ending accuracy of 81%. Participants who were neurologists not epileptologists (n = 14/901) improved by 13% with a mean ending accuracy of 79% whereas epileptologists (n = 32/901) improved by 12% with a mean ending accuracy of 78% (Fig. 3**B**). Break down by all healthcare backgrounds can be found in Supplemental Fig. 1. Participants who reported reading EEGs regularly improved by 12% whereas those who reported not reading EEGs regularly improved by 14%; both groups ended at an accuracy of 81%. (Fig. 3**C**).

**Fig. 4:**
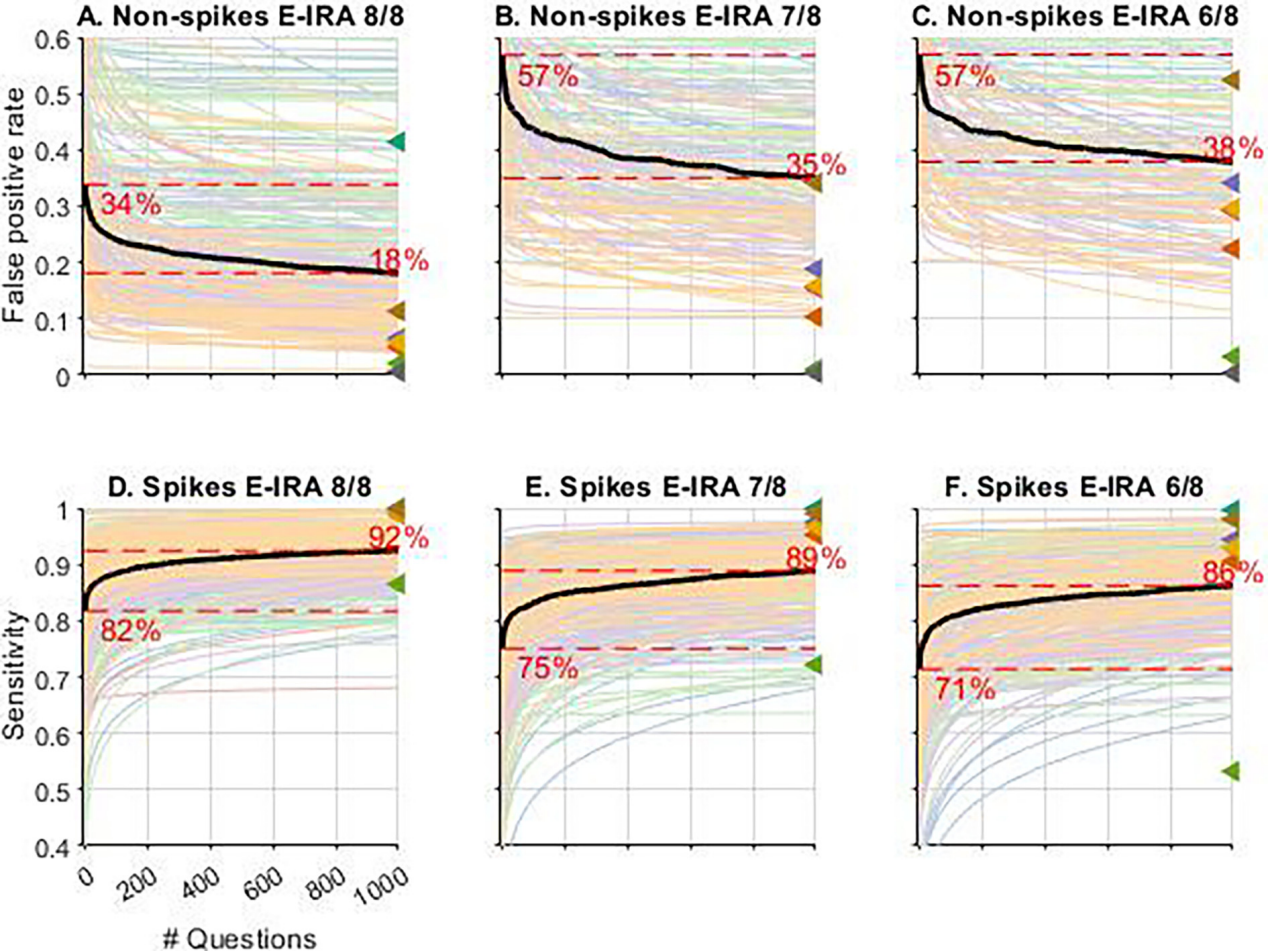
Users’ learning curves stratified by question level of clarity. Legend. “E-IRA” = Expert Interrater Reliability. Candidate IED images were grouped by agreeance of experts to show the learning curve of users for candidate IEDs of different clarities using the same equation used in Fig. 1 (a(n/N)^b^). Colors assigned to users in Fig. 1 based on quantile stayed the same across all plots in Fig. 2. [A, B, C] Show false positive rating assigned by crowd vs the number of questions answered for spikes that received 8/8, 7/8, and 6/8 votes by experts to be non-spikes (clear non-IEDs). [D, E, F] Show sensitivity of ratings assigned by crowd vs the number of question answered for spikes that received 8/8, 7/8, and 6/8 votes by experts to be spikes (clear IEDs).

When questions were grouped according to clarity, we found that users achieved better performance on easier questions both at baseline and throughout training. For the easiest group of non-IEDs, (0/8 votes; Fig. 4**A**), the false positive rate started at 34% and ended at 18% while learning for the slightly more unclear non-IEDs (1/8, and 2/8 votes; Fig. 4**B-C**) started with a false positive rate of 57% (for both 1/8 and 2/8 cases) and ended at 35% (1/8) and 38% (2/8), respectively. The clear IED groups (Fig. 4**D-F**) showed similar trends for sensitivity. The easiest IEDs group (8/8 votes; Fig. 4**D**) started at a sensitivity of 82% and ended at 92%, the group of IEDs that received 7/8 expert votes (Fig. 4**E**) moved from 75% to 89%, and the hardest group of clear IEDs (Fig. 4**F**) moved from 71% to 86%. The results from Fig. 3, Fig. 4 are summarized in Table 1.

**Table 1:** User’s learning rates.

			**Question Difficulty**												**Increase in Accuracy**			
**Quantile at Final Performance**	**a Values**	**b** **Values**	**0 votes** **a Values**	**1 vote** **a** **Value**	**2 votes** **a** **Value**	**6 votes** **a** **Value**	**7 votes** **a** **value**	**8 votes** **a value**	**0 votes** **b Values**	**1 vote** **b** **Value**	**2 votes** **b** **Value**	**6 votes** **b** **Value**	**7 votes** **b** **value**	**8 votes** **b value**	**qn** **1**–**50**	**qn 50**–**200**	**qn 200**–**500**	**qn 500**–**1000**
**All**	**0.85**	**0.02**	**0.87**	**0.71**	**0.68**	**0.96**	**0.93**	**0.91**	**0.02**	**0.03**	**0.03**	**0.02**	**0.02**	**0.02**	**6.61**	**2.47**	**1.67**	**1.29**
**0**–**5**	**0.71**	**0.01**	**0.89**	**0.84**	**0.80**	**0.86**	**0.81**	**0.71**	**0.03**	**0.03**	**0.04**	**0.01**	**0.03**	**0.01**	**3.59**	**1.32**	**0.89**	**0.68**
**5**–**25**	**0.81**	**0.02**	**0.91**	**0.76**	**0.72**	**0.95**	**0.87**	**0.81**	**0.03**	**0.04**	**0.03**	**0.02**	**0.03**	**0.02**	**5.98**	**2.25**	**1.52**	**1.17**
**25**–**50**	**0.88**	**0.03**	**0.87**	**0.75**	**0.71**	**0.96**	**0.92**	**0.88**	**0.02**	**0.03**	**0.03**	**0.02**	**0.02**	**0.03**	**8.51**	**3.26**	**2.23**	**1.73**
**50**–**75**	**0.93**	**0.03**	**0.75**	**0.62**	**0.57**	**0.97**	**0.95**	**0.93**	**0.02**	**0.02**	**0.02**	**0.02**	**0.03**	**0.03**	**8.83**	**3.39**	**2.32**	**1.80**
**75**–**95**	**0.96**	**0.02**	**0.66**	**0.45**	**0.35**	**0.96**	**0.96**	**0.96**	**0.02**	**0.04**	**0.05**	**0.01**	**0.02**	**0.02**	**5.40**	**2.00**	**1.34**	**1.03**

Legend: Users’ learning was modeled using the equation a(n/N)^b^ (a = accuracy at question 5000, N = 5000, b = learning rate, n = question number). The table shows the values for a, b, and incremental accuracy increases for all users stratified in 5 quintile groups (75% − 95%, 50% − 75%, 25% − 50%, 5% − 25%, 0% − 5%) and depending on questions’ level of clarity.

**Fig. 5:**
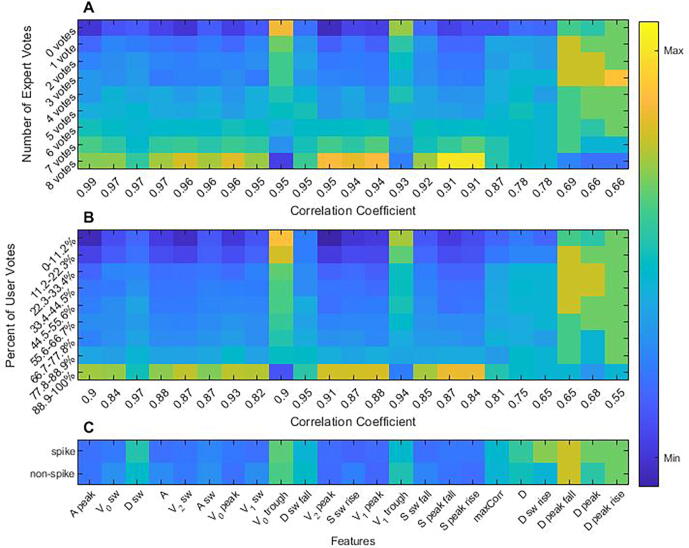
Correlation of candidate interictal epileptiform discharges features and votes by users and experts. Legend. (A) Candidate IEDs were grouped in 9 bins based on number of expert votes. The z normalized median feature value of each bin was plotted by color for all 23 features. (B) The candidate IEDs were regrouped into 9 new bins based on user votes. Matching what was done in plot A, the z normalized median feature value of each bin was plotted by color for all 23 features. (C) The outlier candidate IEDs taken from Fig. 4 were reclassified as IEDs and non-IEDs. The z normalized median feature values were plotted by color for all 23 features of the IEDs and non-IEDs. Feature values in all 3 maps were z normalized using the medians and iqrs for each feature taken from the entire sample of candidate IEDs.

We then investigated the extent to which a small (n = 23) set of well-defined morphological features can account for participant voting behavior. We found that voting behavior for both contest participants and experts were strongly correlated (correlation > 0.9) with values of a core set of 17 of the 23 features (Fig. 5**A-B**).

**Fig. 6:**
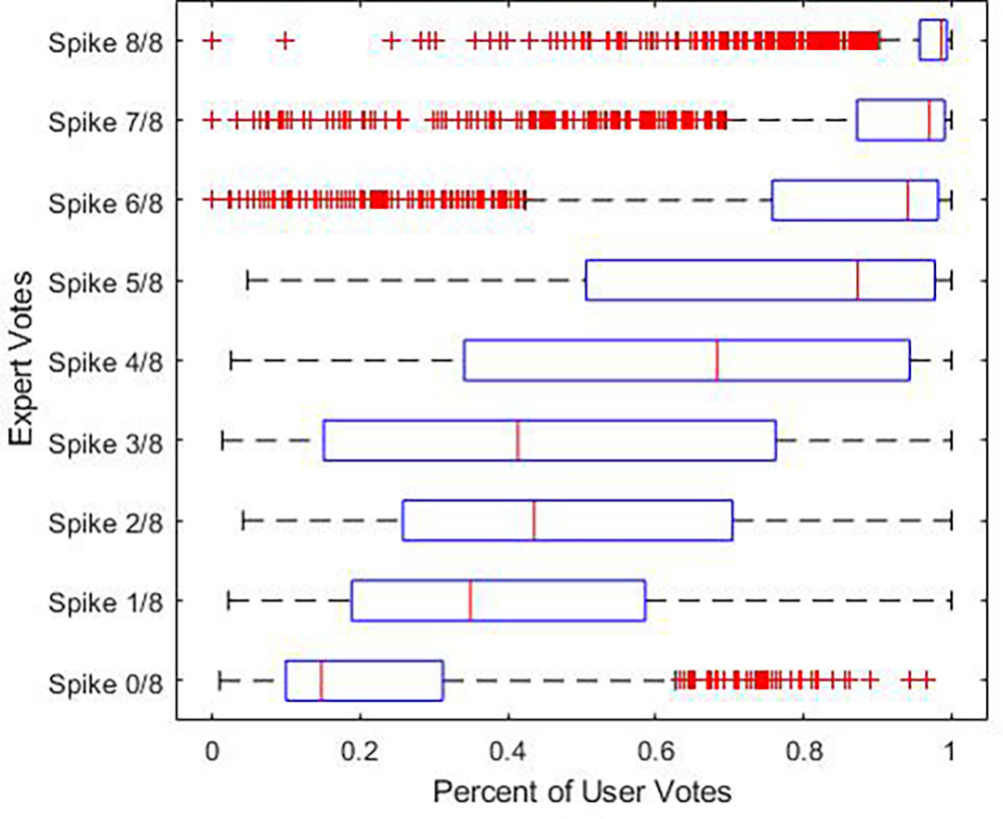
Level of agreement between users and experts. Legend. User and expert rating were compared for each candidate IED by creating a boxplot with experts rating as the y axis and user ratings as the × axis. Users and experts ratings tend to align for candidate IEDs with better expert consensus. Outliers, candidate IEDs that users and experts strongly disagreed on, are marked in red. (For interpretation of the references to color in this figure legend, the reader is referred to the web version of this article.)

While agreement between the percentages of participants and experts voting “yes” for a given candidate IED was generally strong, as shown by the trend of median values in the box plots of Fig. 6, prominent outliers are also evident. These outliers are examples on which the majority of participants strongly disagree with the majority of experts. We found 203 such examples among “clear” IEDs, and 39 such examples among “clear” non-IEDs. We reasoned that either these cases were misclassified by experts but correctly identified by the “wisdom of the crowd”, or that experts possess knowledge of “corner cases” that participants were unable to master during the contest. To distinguish these two possibilities, we performed two analyses.

First, we compared the feature values of these outlier waveforms (Fig. 5**C**). As shown, the median feature values are quite similar for IED and non-IED outlier cases, suggesting that experts rely for these cases on features outside of the features that characterize more typical cases.

Next, three of the authors (JJ, FN, MBW) performed direct visual analysis of all outliers. Examples of outliers are shown in the Supplemental Material. Qualitatively, we grouped outliers based on the special features that may have made them difficult for novices into 2 groups (A and B) and 6 subgroups. Group A included all candidate IEDs rated as non-IEDs by experts but rated as IEDs by users. Group A group was divided into 3 subgroups: (1) no IED inside the indicated red rectangle but IEDs in the vicinity (n = 10), (2) artifacts such as eye blinks, lateral rectus spikes, and myogenic artifact (n = 16), and (3) background activity and sleep structures such as sleep spindles, sharply contoured background activity, and high amplitude slowing (n = 13). Group B consisted of candidates that were classified as IEDs by experts but non-IEDs by users. Group B subgroups included (1) IEDs whose amplitude was low (errors likely associated with users’ inability to change EEG sensitivity in the learning app) (n = 44), (2) IEDs that do not stand out and/or have no well-formed after-going slow wave (n = 53), and (3) IEDs with atypical morphology such as triphasic waves, symmetric up- and down-going slopes, sharply contoured instead of clearly spiky, and sharply contoured slowing (n = 106). We also found that in more than half of the candidate IEDs in group B (n = 122/203) there was pronounced periodicity. We hypothesize that this characteristic helped experts classify these waveforms as IEDs despite the lack of typical features in individual IEDs making up the periodic sequence.

## 4 Discussion

Our competition-based mobile learning application engaged more than 900 participants from around the globe in learning how to recognize IEDs in EEGs. There was no significant difference in user performance upon stratification by profession/level of training (Fig. 3B/Supplemental Fig. S1). This observation may be explained by (i) the relatively lower number of participants who were neurologists or epileptologists and (ii) the overall limited exposure to EEG among students and providers not in the field of neurology, clinical neurophysiology, or epilepsy.

Overall, users who participated improved their accuracy by an average by of 13% (95% CI [0.13, 0.14], p < 0.001, Supplemental Table S2) and achieved a mean ending accuracy of 81%. Although only 10% (n = 89) of users reached expert level (considered to be 90% based on nine experts’ data), our results suggest that rating candidate IEDs in a serial fashion along with instant feedback represents a useful and efficient EEG educational method. Notably, the comparable improvement in performance among experts and those with none-to-minimal prior EEG knowledge may suggest that actually finding sharp transients of interest on EEG (in comparison to rating these sharp transients) is the critical skill to reading EEGs in practice.

Experts and participants seem to use a similar strategy to rate candidate IEDs in terms of analyzing these waveforms’ morphologic features (Fig. 5). While experts and participants are less likely to rely on the duration of the spike and the cross-channel correlation of IEDs, both appear to rely on the area of the peak, duration, voltage, area of the slow wave, and total area of the spike. Nonetheless, we identified a set of candidate IEDs (the outliers) in which experts and participants strongly disagreed upon rating as IED or non-IED. For these examples, the morphologic features of the waveforms that we analyzed appear similar for IEDs and non-IEDs; presumably, experts rely on additional features of the background which non-expert participants did not learn. To identify possible explanations for these outliers, three of the authors (JJ, FN, MBW) performed direct visual analysis of the outlier candidate IEDs. Below we offer qualitative observations from this analysis. Outliers included artifacts (e.g., eye blinks and lateral rectus spikes), background activity especially with a sharp contour, sleep structures, and high amplitude slowing (group A) as well as IEDs with an atypical morphology (e.g., triphasic waves, symmetric epileptiform discharges, and discharges of different configuration such as sharp morphology), with a low amplitude, without an after going slow wave, and those that do not stand out from the background (group B). Importantly, more than half of outliers in group B had a characteristic feature: periodicity.

Understanding the outliers may allow us to optimize future EEG educational materials as well as EEG tests. Based on this data, we advocate that teaching and testing EEG content should include a wide range of artifacts, sharply contoured background activity, sleep structures, discharges with different configurations (e.g., triphasic waves, symmetric discharges, and sharp discharges), discharges without after going slow waves, and discharges that do not prominently stand out from the background. Additionally, our data suggests that periodicity significantly helps experts, classify these candidate IEDs as epileptiform, but that novices did not learn this feature. The concept of periodicity and its association with an epileptiform activity should be transmitted to novices in future versions of the learning application.

Interestingly for 23% (n = 56) of the outlier waveforms, the three authors agreed with the verdict arising from the crowd instead of the verdict of the original 8 gold standard experts. We believe that these examples likely arose from rare instances (0.4% of all cases) where the majority of experts mis-rated candidate IEDs due to noise inherent in the task (e.g. attributable to fatigue, or keyboard error, as some errors are likely unavoidable even by experts when annotating > 13 K events). In contrast, the crowd – given its larger size, and despite being “noisier” – would rate these waveforms correctly. These observations may suggest that crowdsourcing may sometimes be an option for obtaining an accurate gold standard in rating IEDs on EEG, or for flagging instances of “label noise” (expert error) within an expert-labeled gold standard.

Through crowdsourcing, we obtained answers for each candidate IED with an accuracy of 84%. Notably, this figure lies between the mean start (81%) and end (90%) accuracy of the A9 experts used in this study, suggesting that combining votes from a large (N = 901) crowd of participants of mixed experience was nearly as accurate as a relatively small (N = 9) group of experts.

Investigating optimized methods to teach EEG is crucial because accurate EEG interpretation plays a major role in the care of patients with seizures and epilepsy. In select circumstances, having an EEG with IEDs after a single unprovoked seizure warrants the diagnosis of epilepsy and antiseizure treatment initiation (Fisher et al., 2014). Despite its importance in epilepsy care, EEG misinterpretation and resultant epilepsy misdiagnosis is still common in current American practice (Benbadis et al., 2004). This observation has been linked with the fact that a large portion of EEGs in the U.S. are read by neurologists without fellowship training in clinical neurophysiology (Benbadis, 2010), most of whom receive suboptimal EEG education during residency training (Nascimento et al., 2020). Corroborating evidence was presented in a study where academic neurologists and neurologists with clinical neurophysiology or epilepsy certification performed significantly better at IED identification than private practice neurologists and those without these board certifications (Halford et al., 2018).

In this context, attention has been shifted towards scrutinizing neurology residency EEG education. A recent survey of in-residency EEG education in the U.S. focused on residency program directors showed that there is an overall lack of consistency in EEG teaching and evaluation (Nascimento and Gavvala, 2021). Barriers have been reported to be associated with minimal time devoted to EEG during training (mean of 1.7 months) and insufficient EEGs reviewed per resident as a result (40 or fewer studies in two-thirds of participating programs) (Nascimento and Gavvala, 2021). We believe that novel EEG education methods such as the app-based competition approach reported herein may address both above-mentioned barriers. Our app-based training has the advantage that it condenses a high number of EEG samples with candidate IEDs and delivers focused, on-demand teaching on how to identify epileptiform discharges to trainees in a relatively short period of time. Based on our data, users on average completed 100 questions in less than 6 min. An additional barrier reported by program directors is that most residency programs in the U.S. do not utilize objective measures to assess EEG milestones, including the ones proposed by the ACGME (Kash et al., 2009, Nascimento and Gavvala, 2021), and lack clearly established requirements for successful completion of an EEG rotation. Our app-based training may also address these issues since it can be used as an evaluation tool if its instant feedback function is deactivated. Programs may consider making it a pre-requirement for completing an EEG rotation to achieve a certain minimum level of accuracy on our application.

In addition to the high number of candidate IEDs presented to participants in a relatively short period of time, we believe that its instant feedback function is this method’s main educational driving force. As noted by a recently proposed multi-theories learning model (Taylor and Hamdy, 2013), the feedback phase of learning is crucial to effective learning. It should be noted, however, that participants with prior consolidated EEG knowledge may benefit more from such a feedback-based learning tool. These learners, in comparison to those without any prior EEG knowledge, would improve their skills based upon receiving instant feedback and being able to consolidate their knowledge. Notably, we are unable to compare the effectiveness of our method with other teaching platforms due to the lack of other EEG educational interventions focused on IED identification and based on immediate feedback. Nonetheless, teaching a small group of neurology trainees how to rate candidate IEDs using the operational criteria to define IEDs proposed by the International Federation of Clinical Neurophysiology (Kane et al., 2017) resulted in statistically significant improvement in accuracy from 64% to 81% (Kural et al., 2022). We believe that our app-based tool would benefit from being used alongside a preceding strong educational session delivering the basics of IED identification (Kural et al., 2020). The combination of an educational session and our app-based practice would likely benefit all participants irrespective of their prior knowledge given that this approach would address all learning stages: dissonance, refinement, organization, feedback, and consolidation (Kural et al., 2020).

Our study has important limitations. First, whereas the original 8 experts who set the gold standard could modify montages, adjust sensitivity, and analyze the data on a computer screen, in the learning app users could not modify the images, and viewed data on a phone screen, which is generally smaller. The inability to manipulate EEG settings/filters/montages and decreased screen size might have negatively influenced users’ performance. Second, we defined “positive” examples for feedback purposes as those that received 3 or more of the original 8 experts’ votes. This threshold was a determined by consensus among the authors considering the inherent tradeoff between sensitivity and false positive rates associated with varying thresholds. Nevertheless, our results demonstrate the effectiveness of the phone-app approach to learning, a result that does not depend critically on the choice of threshold. Third, participants were not required to use the app in a uniform way; users could complete questions all at once or over multiple sessions. Multiple sessions may result in higher long-term retention of knowledge (Nascimento et al., 2022). Additionally, our study did not assess medium-to-long-term knowledge retention in rating candidate IEDs. Fourth, our subgroup analysis – based on users’ background information including profession and regularity of EEG reading in practice – was limited because there were few users who were classified as neurologists or epileptologists in comparison to non-neurologists/non-epileptologists. Similarly, there were relatively few users who reported reading EEG regularly in comparison to those who reported not doing so. The sample sizes possibly explains the inability of our study to detect potential differences in performance and levels of improvement among non-epileptologists neurologists vs. epileptologists. There is a possibility that our app-based training benefits some users more than others based upon their background EEG knowledge; however, this study was not able to address this particular question.

In addition to these limitations, we have identified areas in which this learning model may be improved. One item is that the ability to find sharp transients of interest on EEG was not taught or assessed in our educational tool. Another limitation derives from the fact that all but one of the original 8 experts and 4 of the additional 9 (A9) experts either trained or currently practice at the same institution (Massachusetts General Hospital). This may represent a bias where these experts may share same reasoning upon rating candidate IEDs on EEG. Moreover, our study was not designed to measure potential improved clinical outcomes derived from users’ improvement in EEG performance. Further, our study primarily addressed recognition of IEDs in the outpatient setting. Future work and other teaching tools are needed to help trainees become proficient at recognizing epileptiform patterns in the intensive care unit and emergency settings. In addition, a high performance in our app-based teaching resource does not necessarily reflect competency in reading EEG in clinical practice. In the latter, for example, readers would need to be proficient in finding sharp transients of interest in EEG – such skill, as discussed above, was not taught or assess in our educational tool. Lastly, our expert consensus-based gold standard was grounded in expert experience rather than an externally validated and objective source such as defining IEDs as those sharp transients seen in patients with video-EEG-confirmed epilepsy. Notably, each of these two gold standards holds advantages and disadvantages hence they should ideally complement each other^15^.

In summary, our app-based training created massive engagement around the world, and improved participants’ accuracy in identifying interictal epileptiform discharges. Given the high density of candidate IEDs included in the training, coupled with the app’s inherent flexibility in allowing users to practice in their own environment and on their own time, this educational method may be especially valuable for neurology trainees as well as healthcare providers with minimal-to-none prior EEG knowledge. We believe that the educational impact of this training can be further increased by adding formal focused didactics before users engage in rating candidate IEDs. Further educational studies should include other types of EEG content (e.g., other findings such as slowing as well as ICU EEG), analyze users’ performance depending on their prior level of EEG knowledge, and assess the feasibility of implementing app-based EEG teaching in residency and fellowship training.

## Author contributions

**Table:** 

**Name**	**Location**	**Role**	**Contribution**
Jaden D. Barfuss	Massachusetts General Hospital, Boston, MA.	Author	Conceptualized and designed study, analyzed and interpreted data, drafted manuscript.
Fábio A. Nascimento, MD	Massachusetts General Hospital, Boston, MA.	Author	Conceptualized and designed study, analyzed and interpreted data, drafted manuscript.
Erik Duhaime	Centaur Labs, Boston, MA.	Author	Conceptualized and designed study, played a major role in acquisition of data, reviewed manuscript.
Srishti Kapur	Centaur Labs, Boston, MA.	Author	Conceptualized and designed study, played a major role in acquisition of data, reviewed manuscript.
Loannis Karakis, MD	Emory University School of Medicine, Atlanta, GA.	Author	Played a major role in creating gold standard.
Marcus Ng, MD	University of Manitoba, Winnipeg, MB, Canada.	Author	Played a major role in creating gold standard.
Aline Heropian, MD	Yale University, New Haven, CT.	Author	Played a major role in creating gold standard.
Alice Lam, MD	Massachusetts General Hospital, Boston, MA.	Author	Played a major role in creating gold standard.
Douglas Maus, MD, PhD	Massachusetts General Hospital, Boston, MA.	Author	Played a major role in creating gold standard.
Jonathan J. Halford, MD	Medical University of South Carolina, Charlestown, SC.	Author	Played a major role in creating gold standard.
Sydney Cash, MD, PhD	Massachusetts General Hospital, Boston, MA.	Author	Played a major role in creating gold standard.
M. Brandon Westover, MD, PhD	Massachusetts General Hospital, Boston, MA.	Author	Conceptualized and designed study, analyzed and interpreted data, reviewed manuscript, supervised study.
Jin Jing, PhD	Massachusetts General Hospital, Boston, MA.	Author	Conceptualized and designed study, analyzed and interpreted data, reviewed manuscript, supervised study.

## Declaration of Competing Interest

The authors declare the following financial interests/personal relationships which may be considered as potential competing interests: E. Duhaime and S. Kapur of Centaur Labs developed and have a financial interest in the DiagnosUs app used to collect the data. J. Barfuss, F. Nascimento, I. Karakis, M. Ng, A. Herlopian, A. Lam, D. Maus, J. Halford, S. Cash, M. Westover, and J. Jing report no disclosures.
