# Supplementary material for: On-demand EEG education through competition – A novel, app-based approach to learning to identify interictal epileptiform discharges
Source: Clin Neurophysiol Pract. 2023 Aug 19;8:177–86. doi: 10.1016/j.cnp.2023.08.003 (PMC10480673; doi:10.1016/j.cnp.2023.08.003)

Supplemental Material

Supplemental Table 1: Background of Participants

| Background | Physicians (MD/DO) | Nurse Practitioners | Physician Assistants | Medical Students | Healthcare Students | Other Professions | Unknown |
| --- | --- | --- | --- | --- | --- | --- | --- |
| # of participants | 82 (9%) | 40 (4%) | 14 (2%) | 238 (26%) | 146 (16%) | 322 (36%) | 59 (7%) |
|  | 16 Neurologists  32 Epileptologists | |  |  |  |  |  |

Legend: Break down of user demographics by medical profession.

Supplemental Table 2: T-Test Q1→Q1000

Paired Samples Statistics

|  | Mean | N | Standard Deviation | Standard Error Mean |
| --- | --- | --- | --- | --- |
| Accuracy at Q1 | 0.6745 | 901 | 0.09771 | 0.00326 |
| Accuracy at Q1000 | 0.8114 | 901 | 0.06980 | 0.00233 |

Paired Samples Test

|  |  |  | 95% Confidence Interval of the Difference | |  |  | Significance | |
| --- | --- | --- | --- | --- | --- | --- | --- | --- |
| Mean | Standard Deviation | Standard Error Mean | Lower | Upper | t | df | One-Sided P | Two-Sided P |
| 0.13689 | 0.0993 | 0.00331 | 0.1304 | 0.14339 | 41.381 | 900 | <0.001 | <0.001 |

Legend: Statistical analysis performed using IBP (SPSS) Statistics Version 28.0 software.


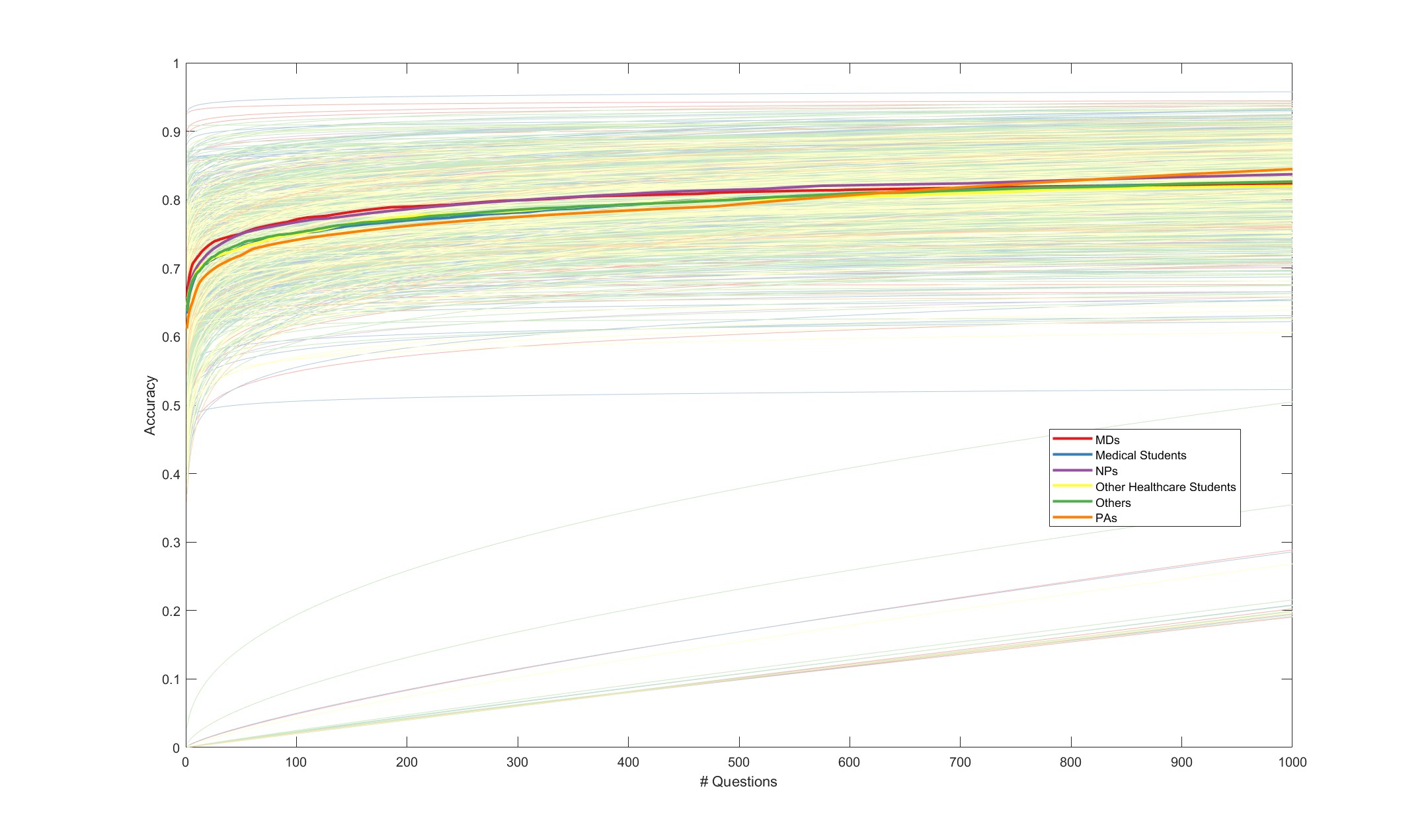
Supplemental Figure 1: Users performance by profession

Legend: Performance for each user was graphed and colored by profession. Solid bright colored lines represent the median performance of each profession.

Legend: User learning based on medical perfession. Dark solid lines represent the average performance of each group.

Supplemental Figure 2: Expert Accuracy (Additional 9) vs. Years of Experience


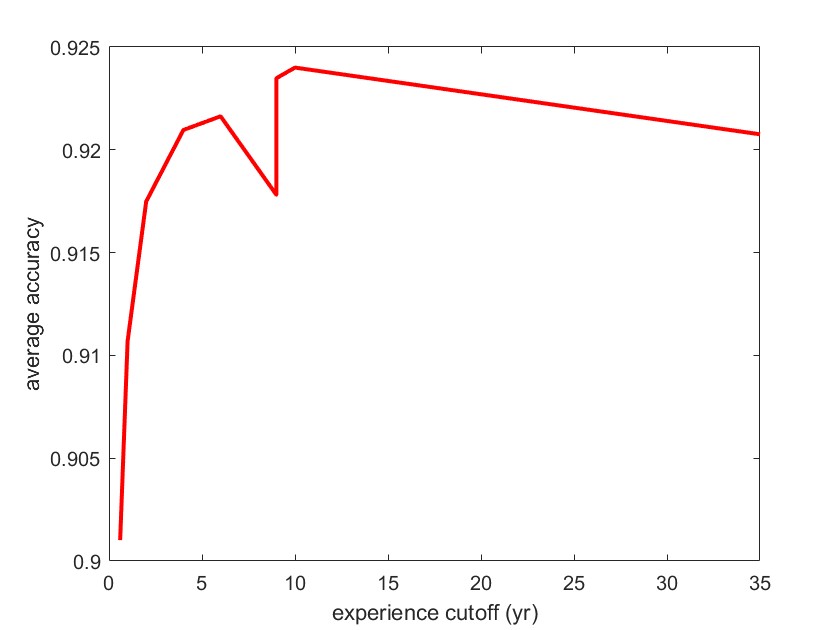


Legend: Average expert Accuracy remains between 90 – 92.5% regardless of what experience level choose to set the cutoff at for years of experience to be included in the Additional 9 used to create the expert level.

6 examples of outliers (average)
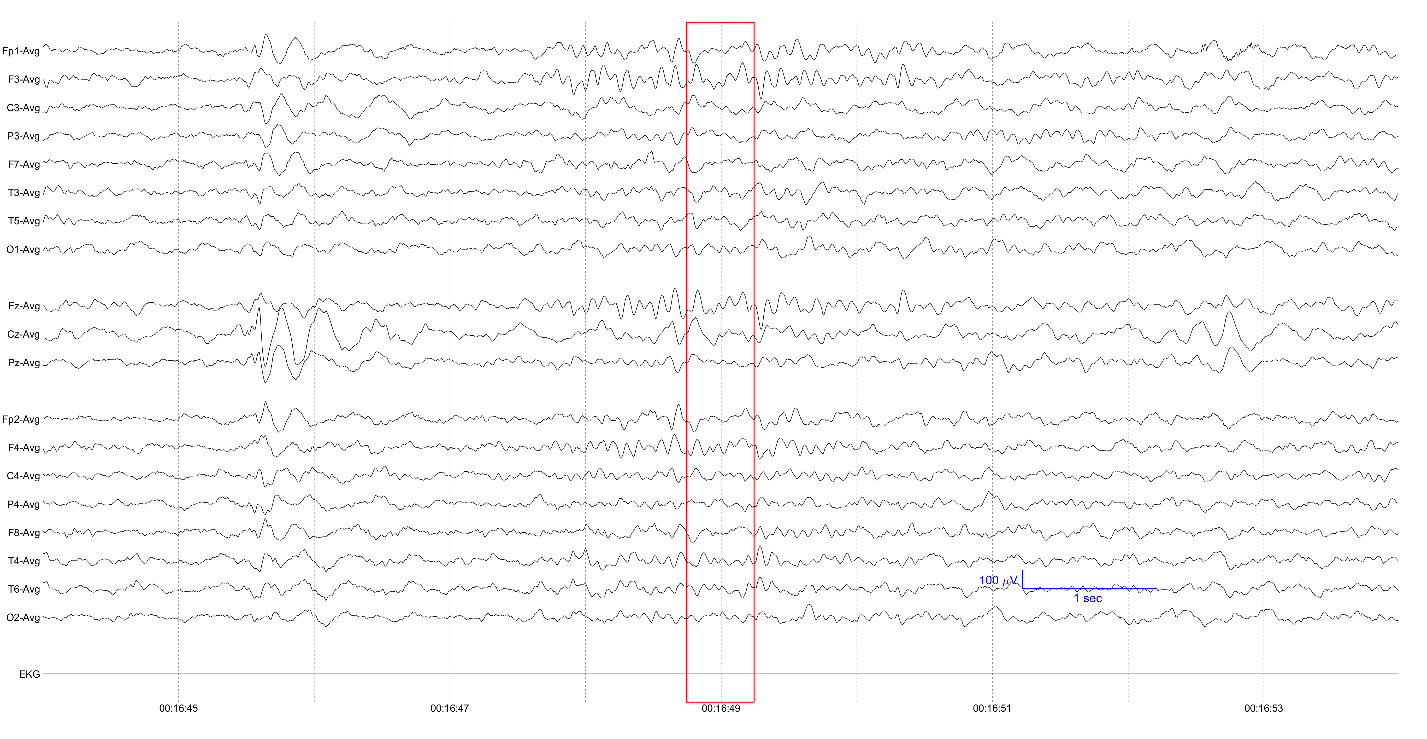

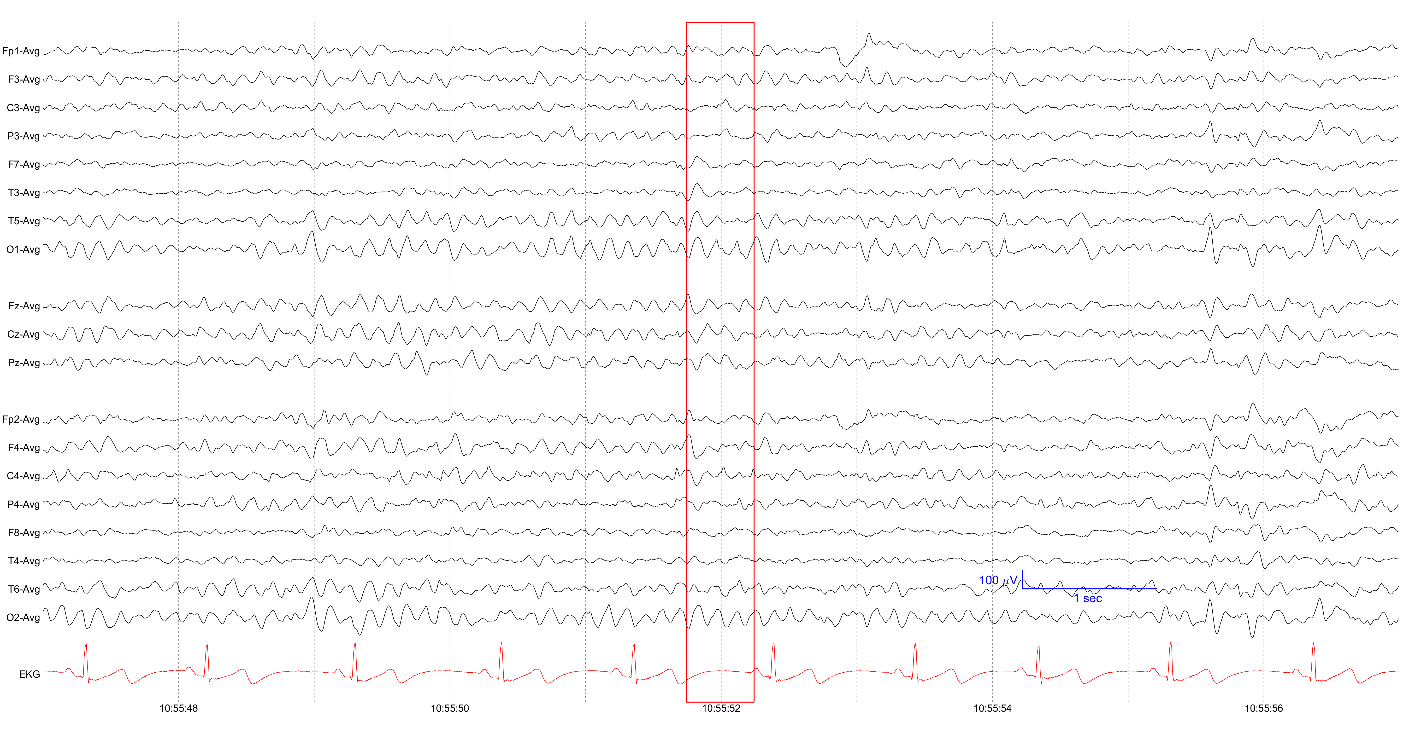

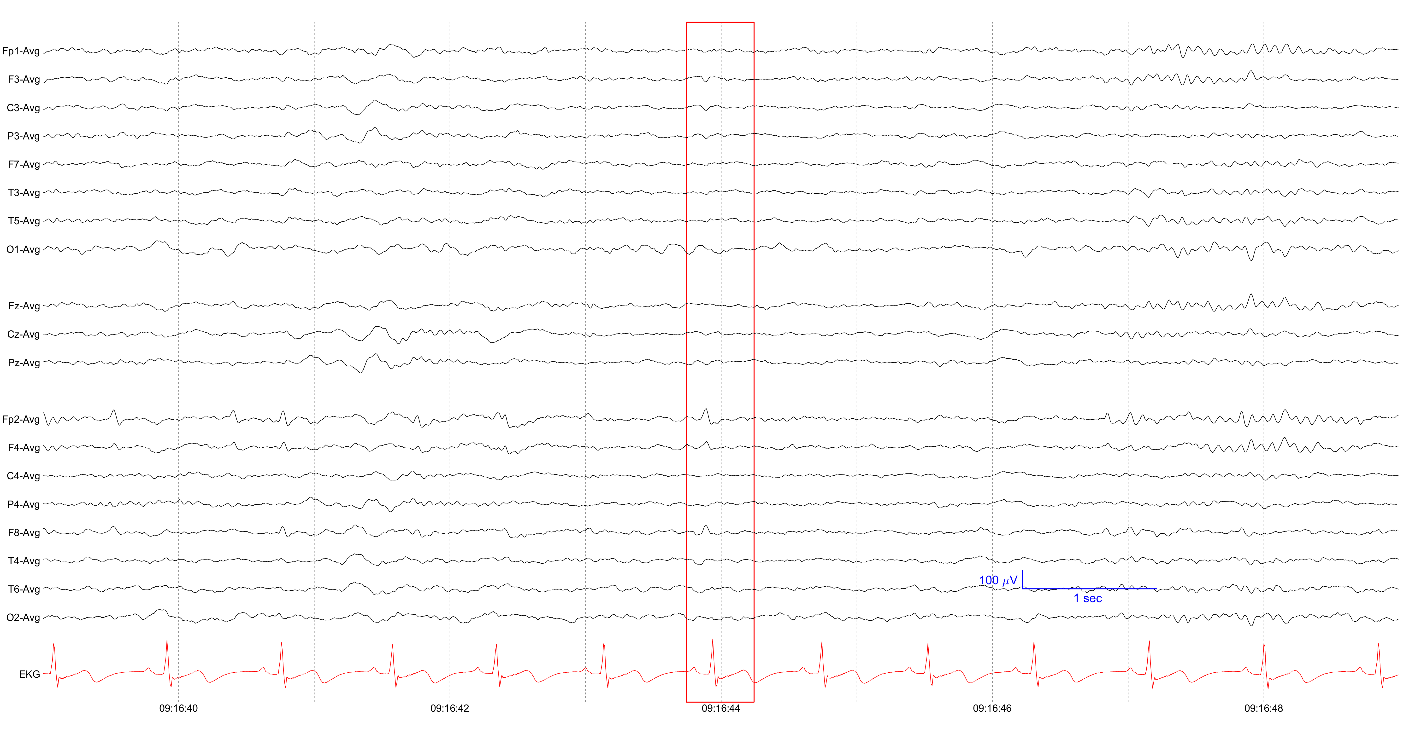

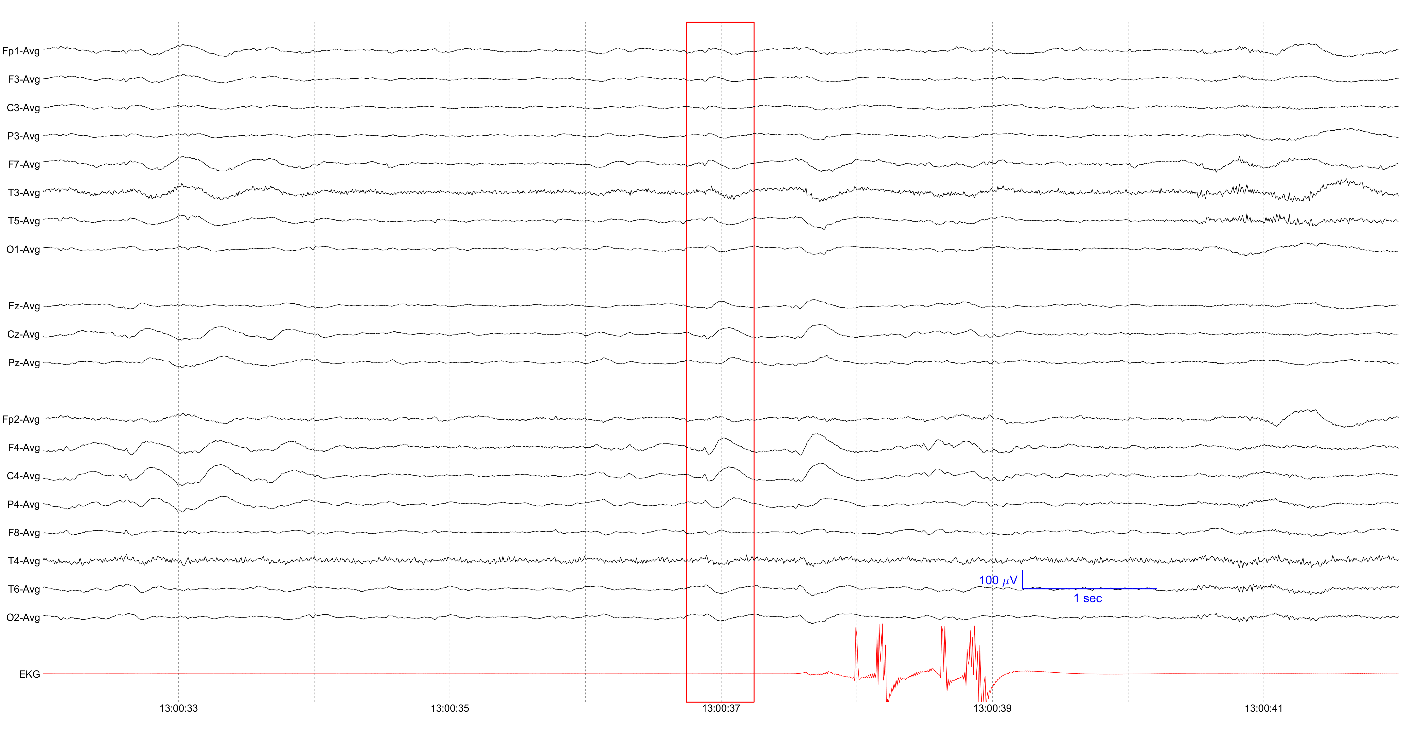


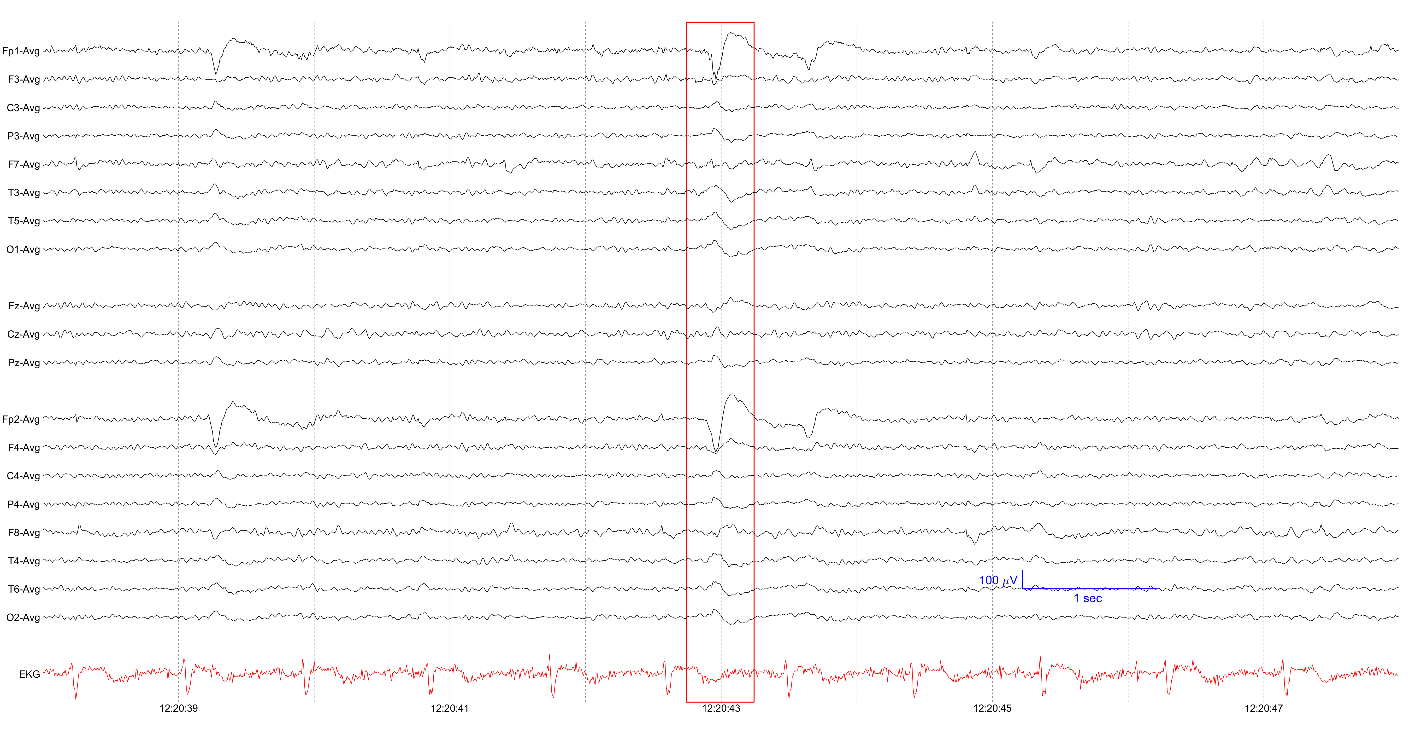


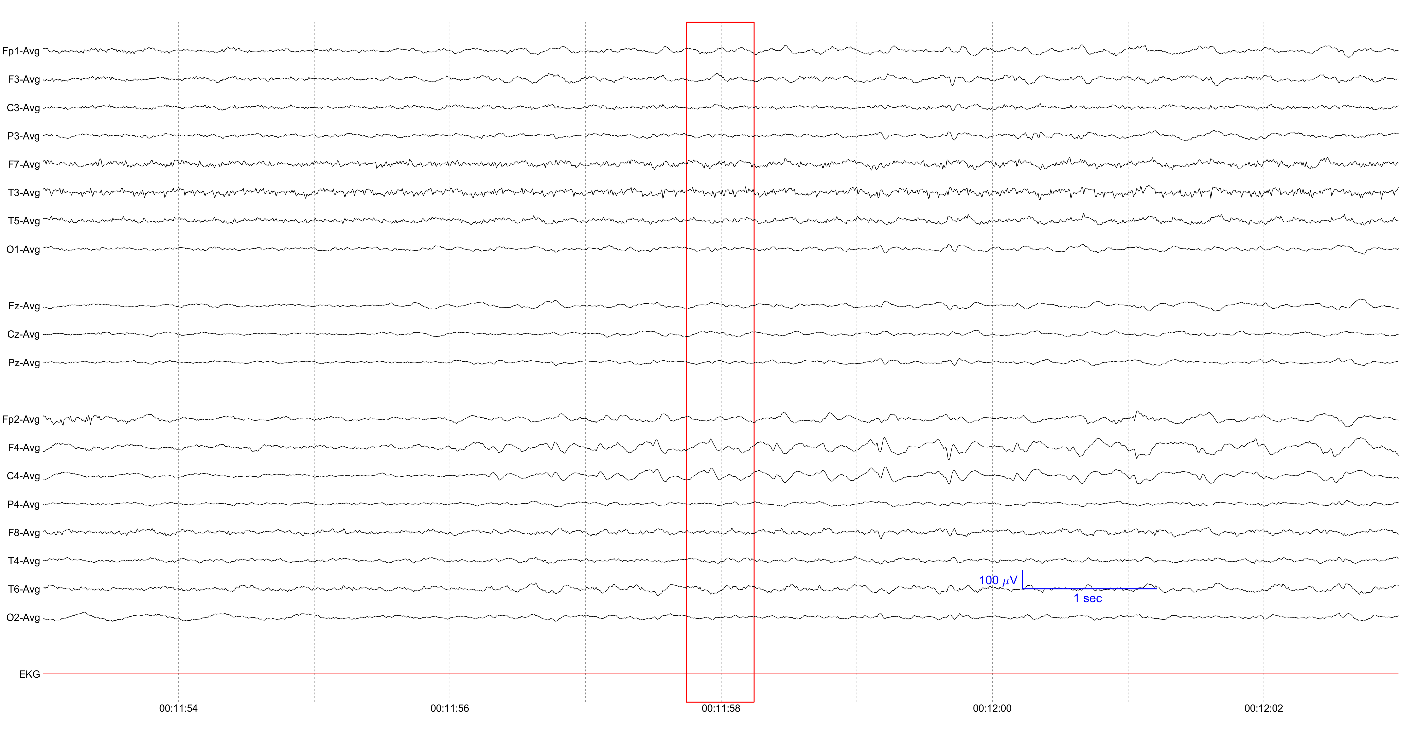


5 examples IED (bipolar/average)


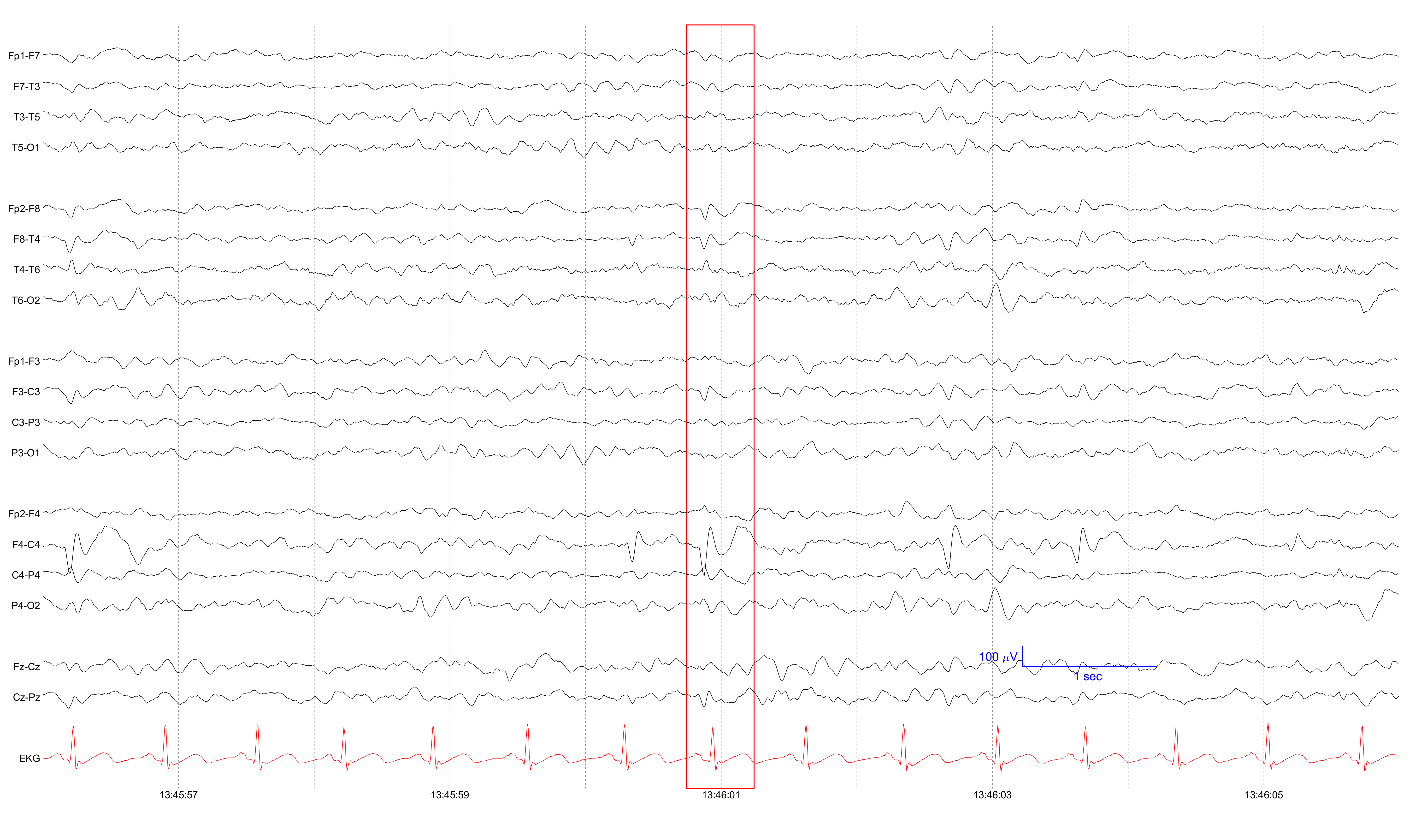

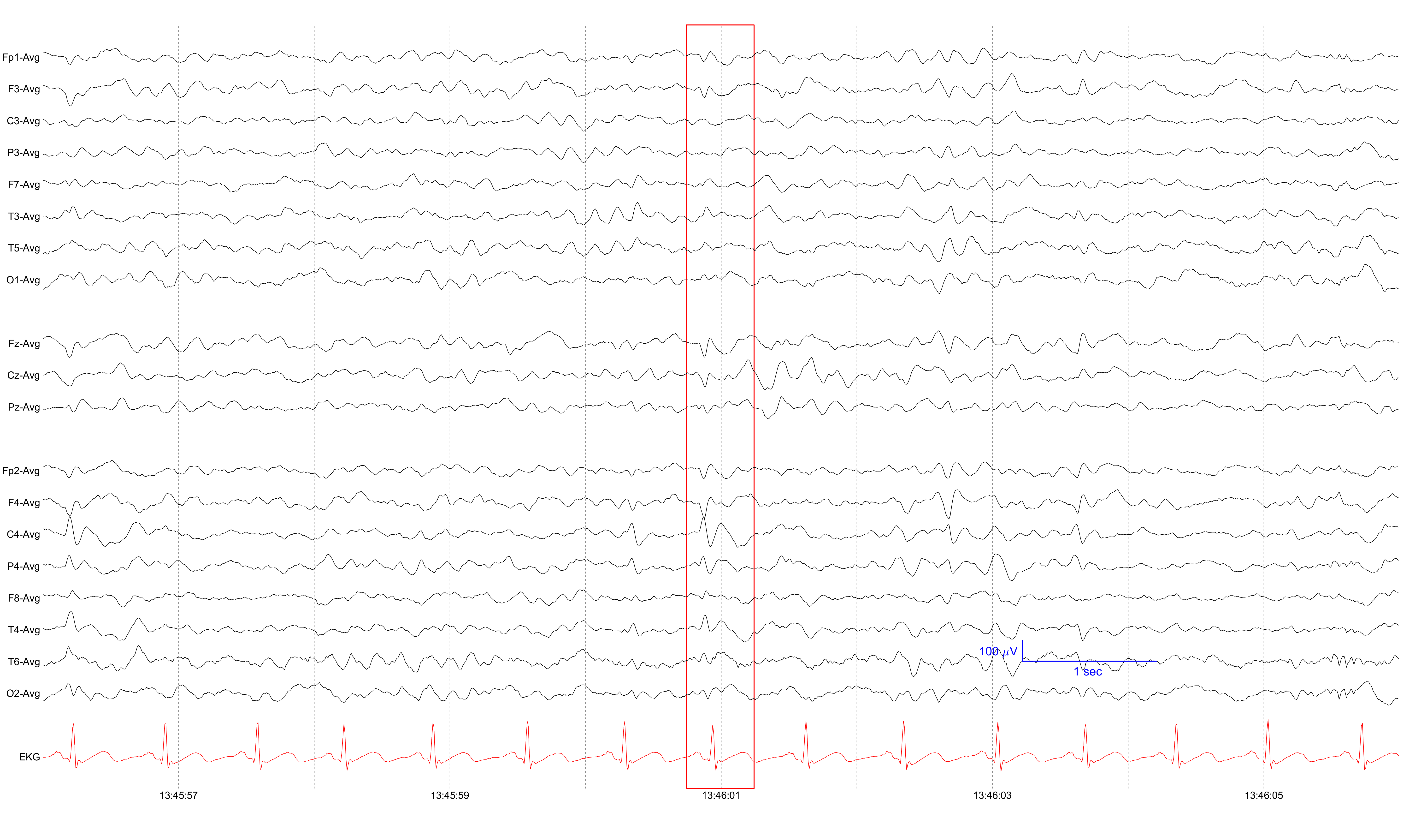

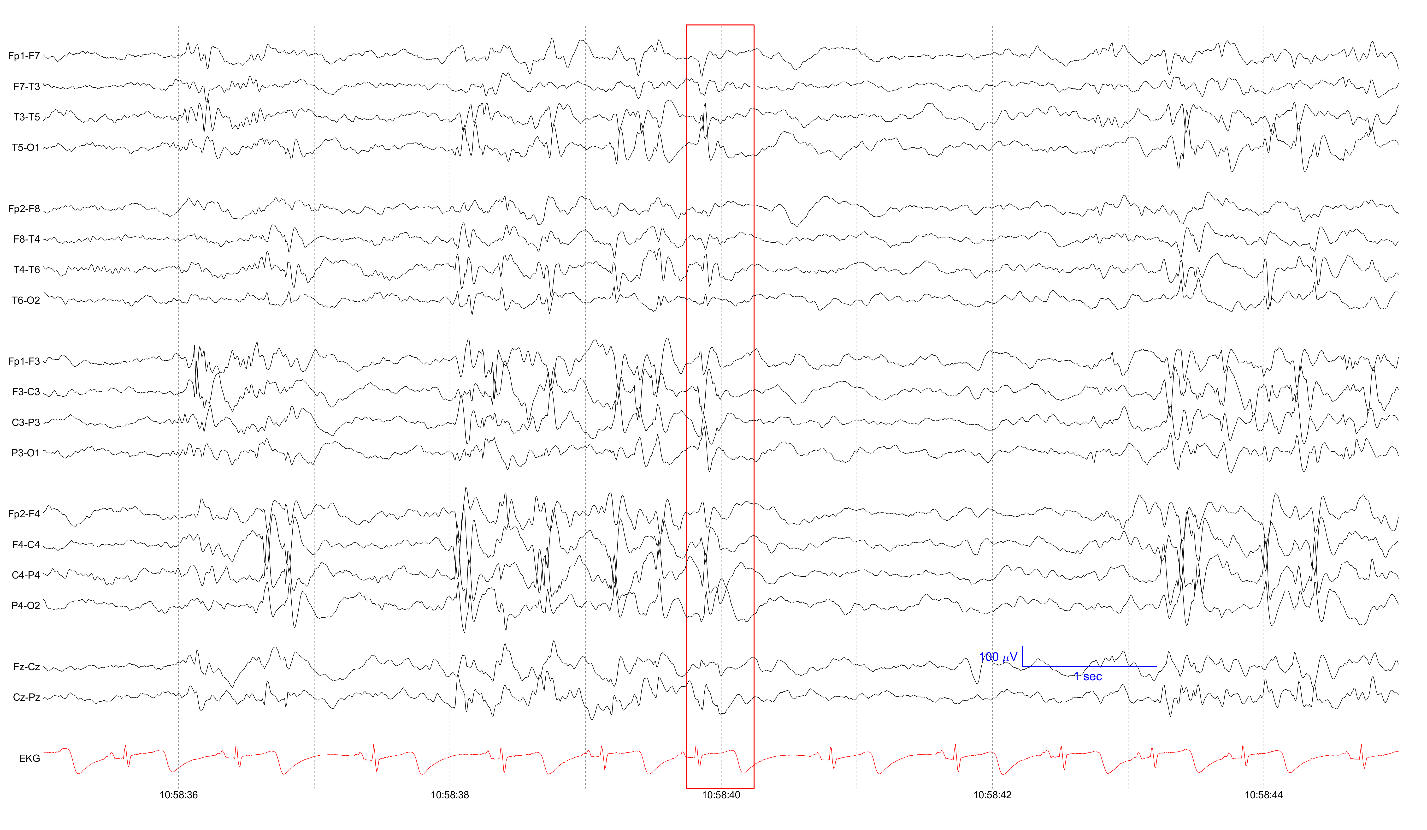

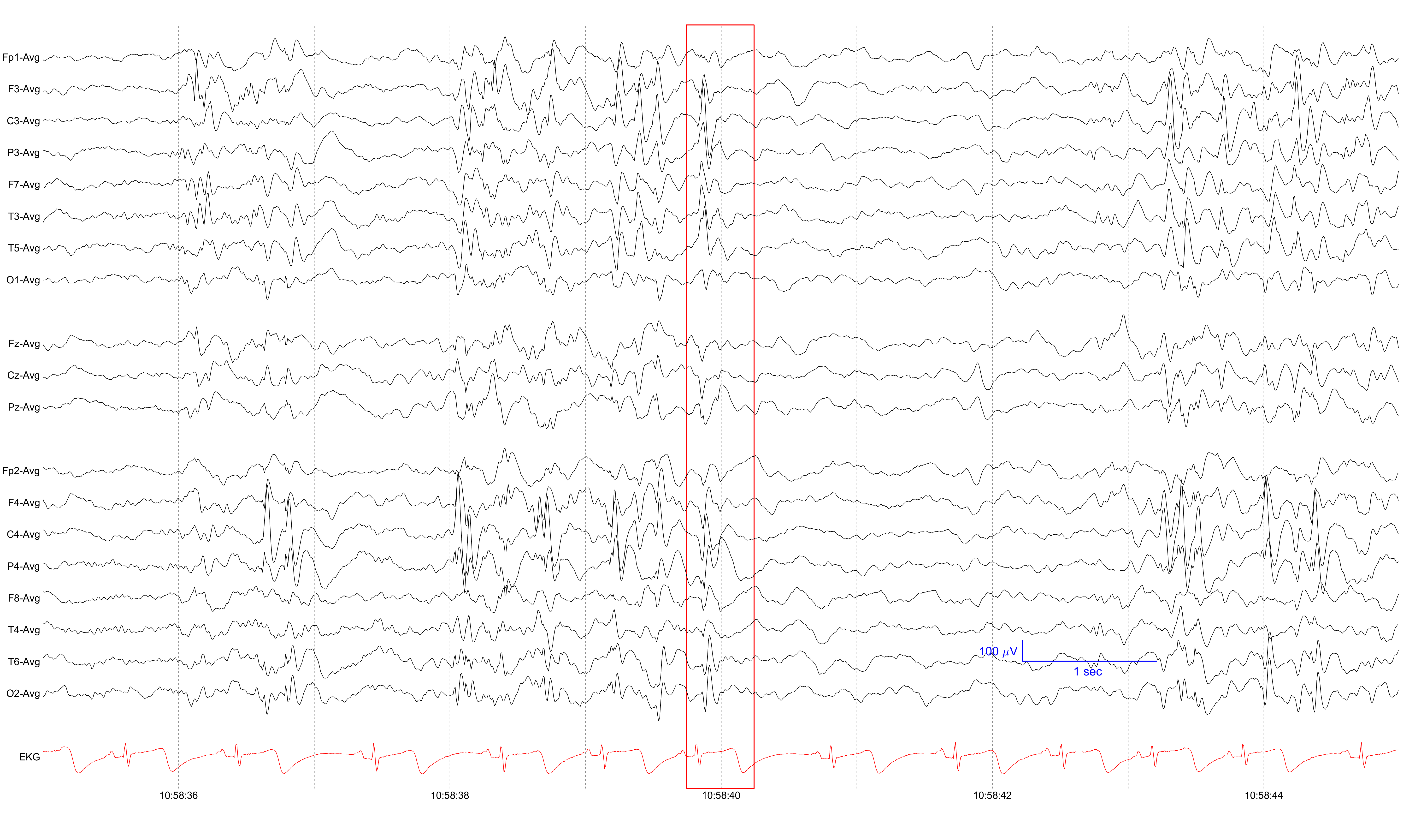

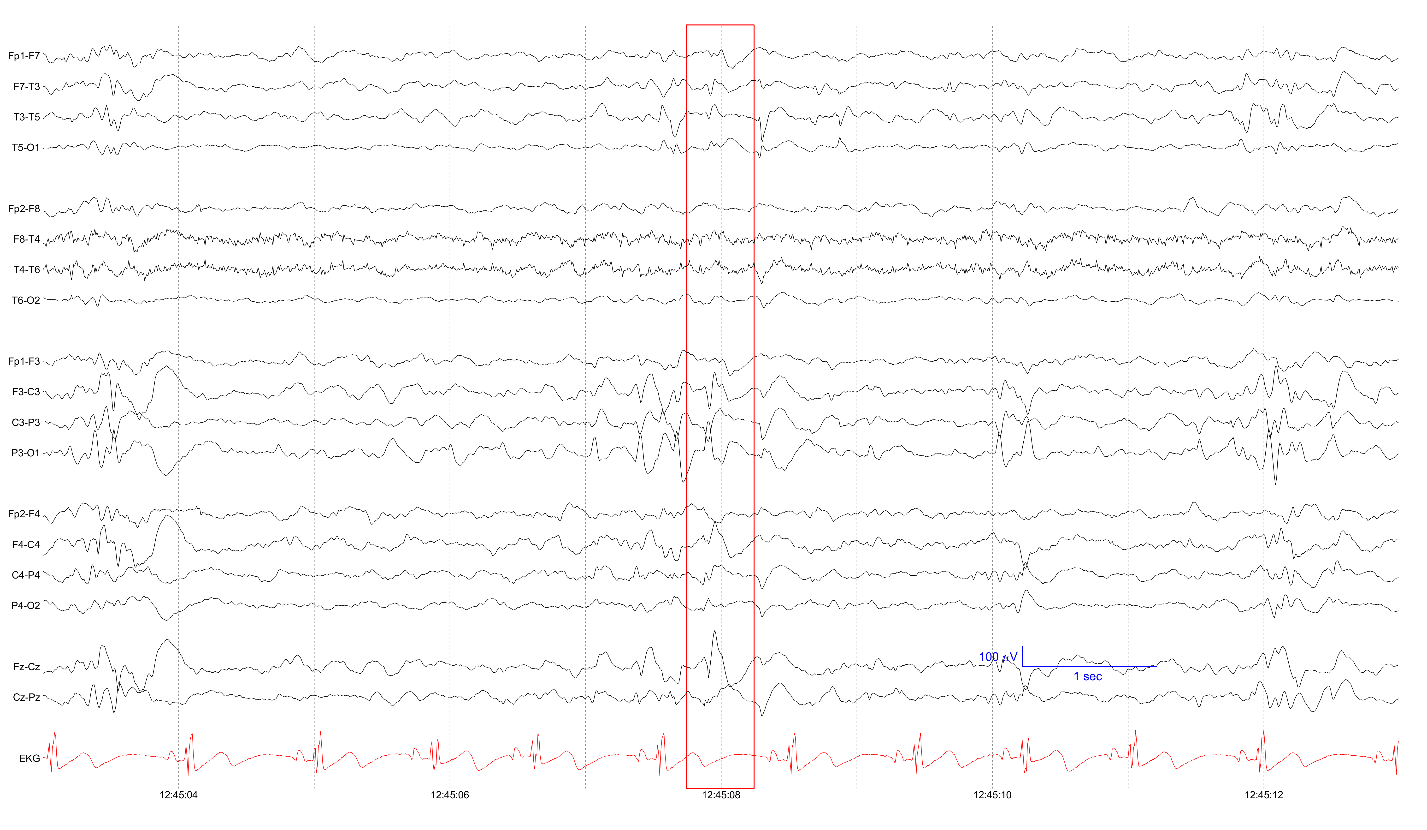

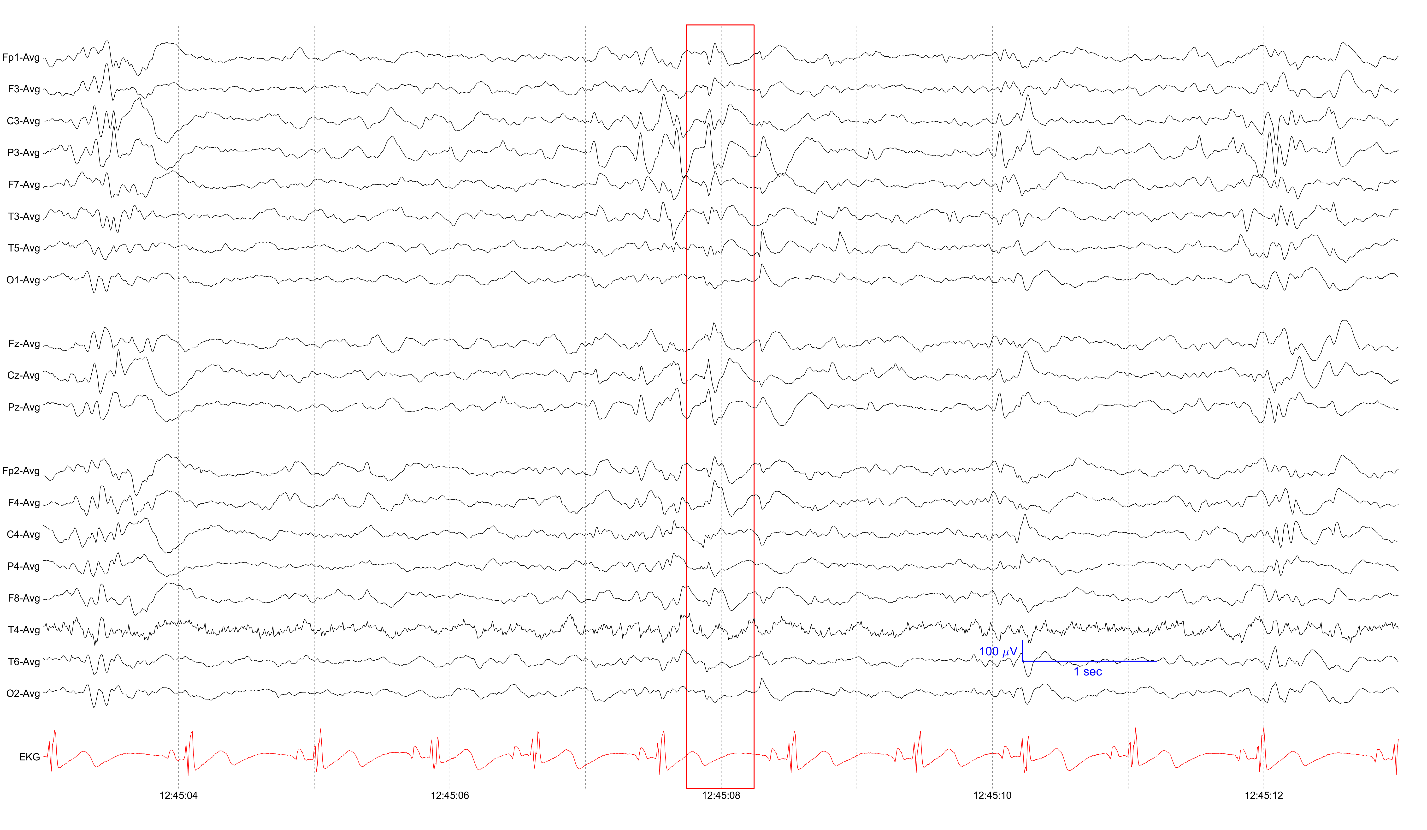

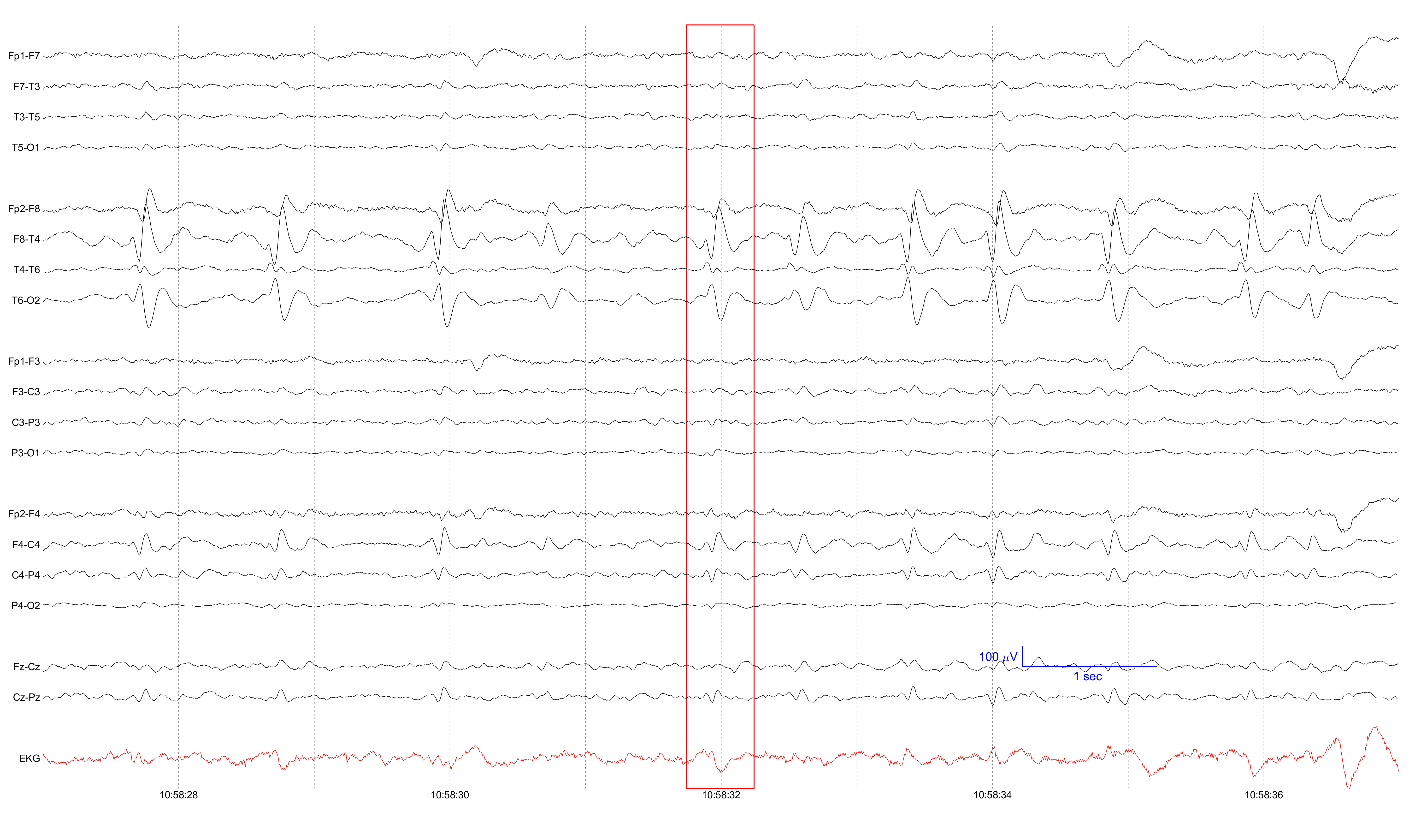

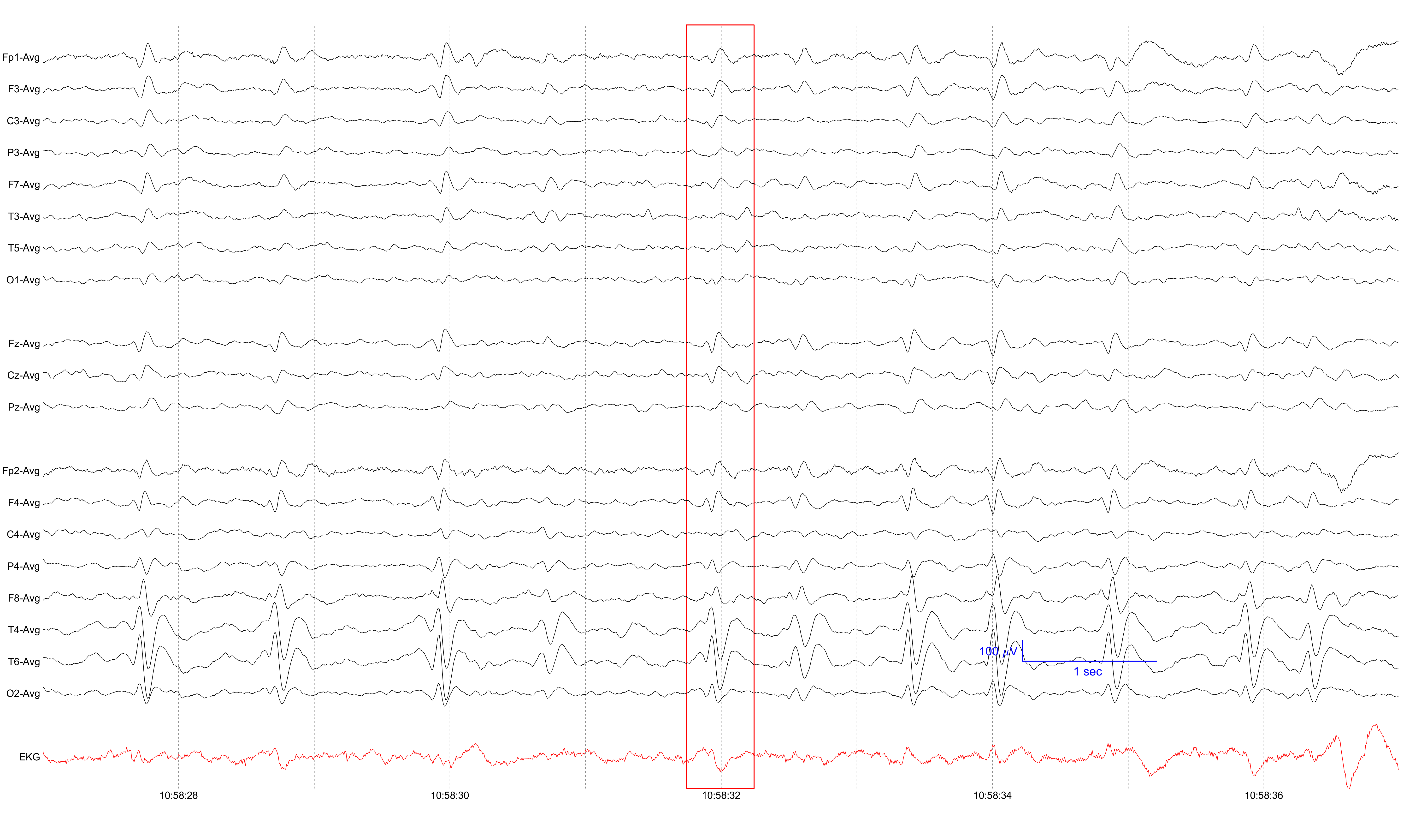

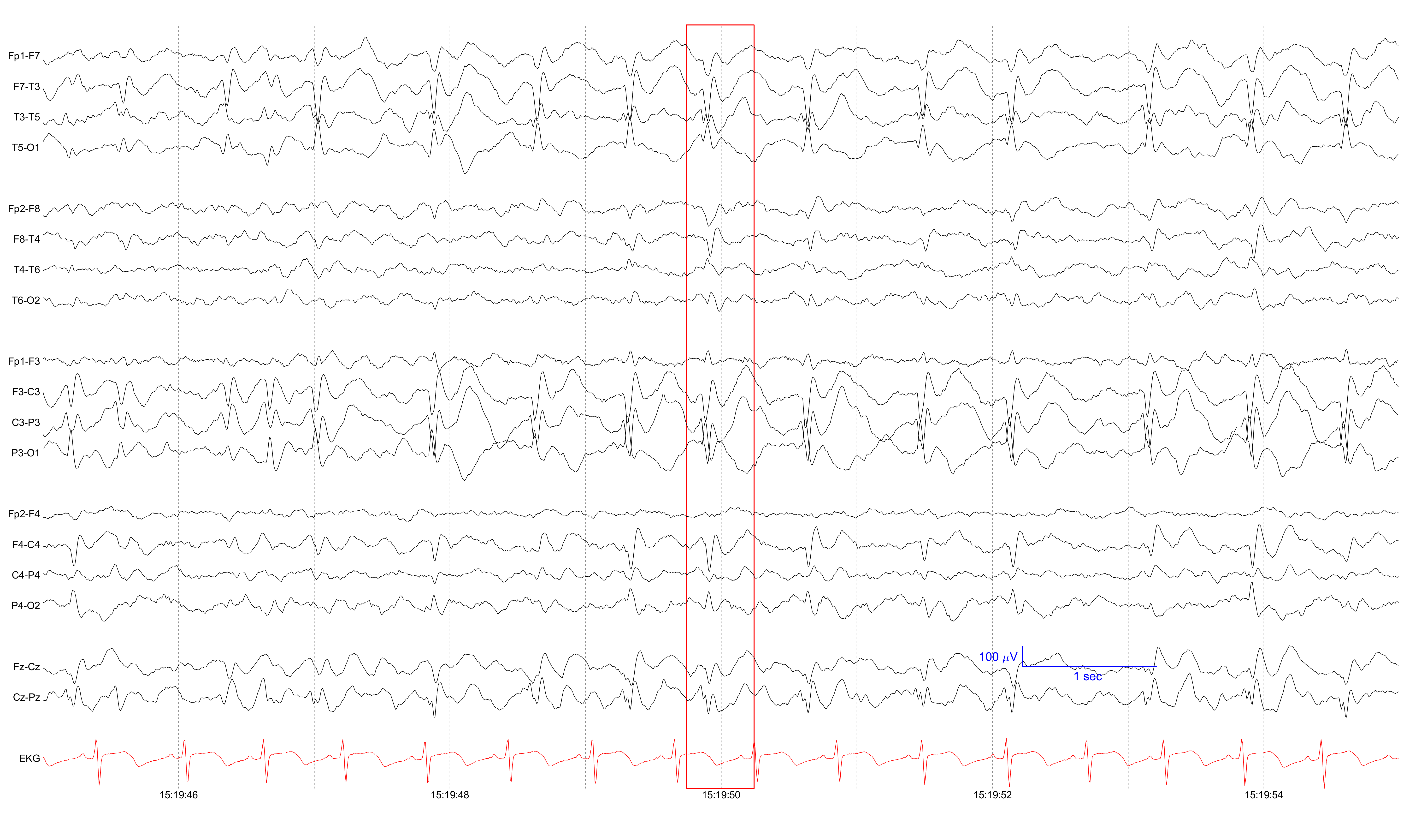

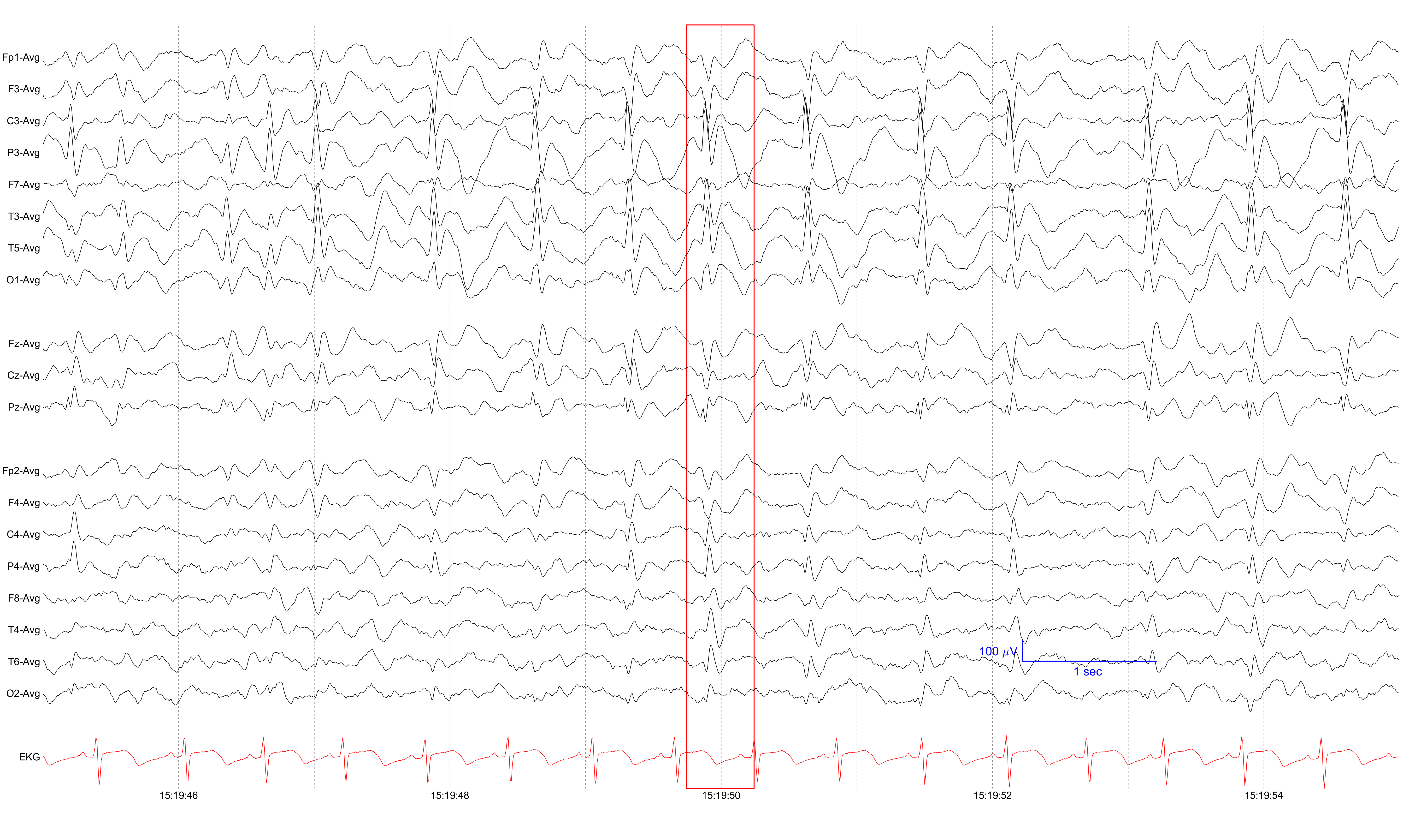


5 examples non-IED (bipolar/average)


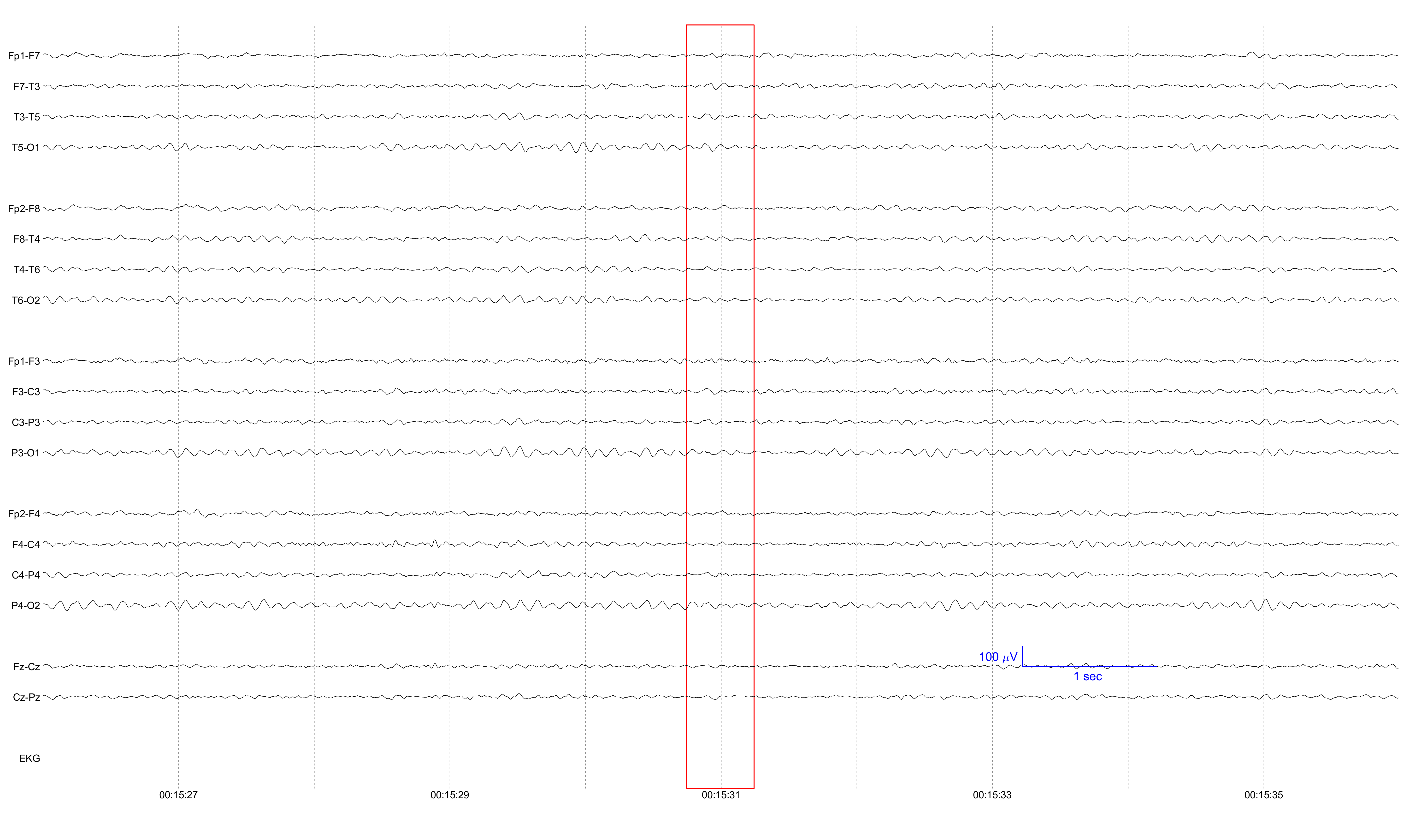

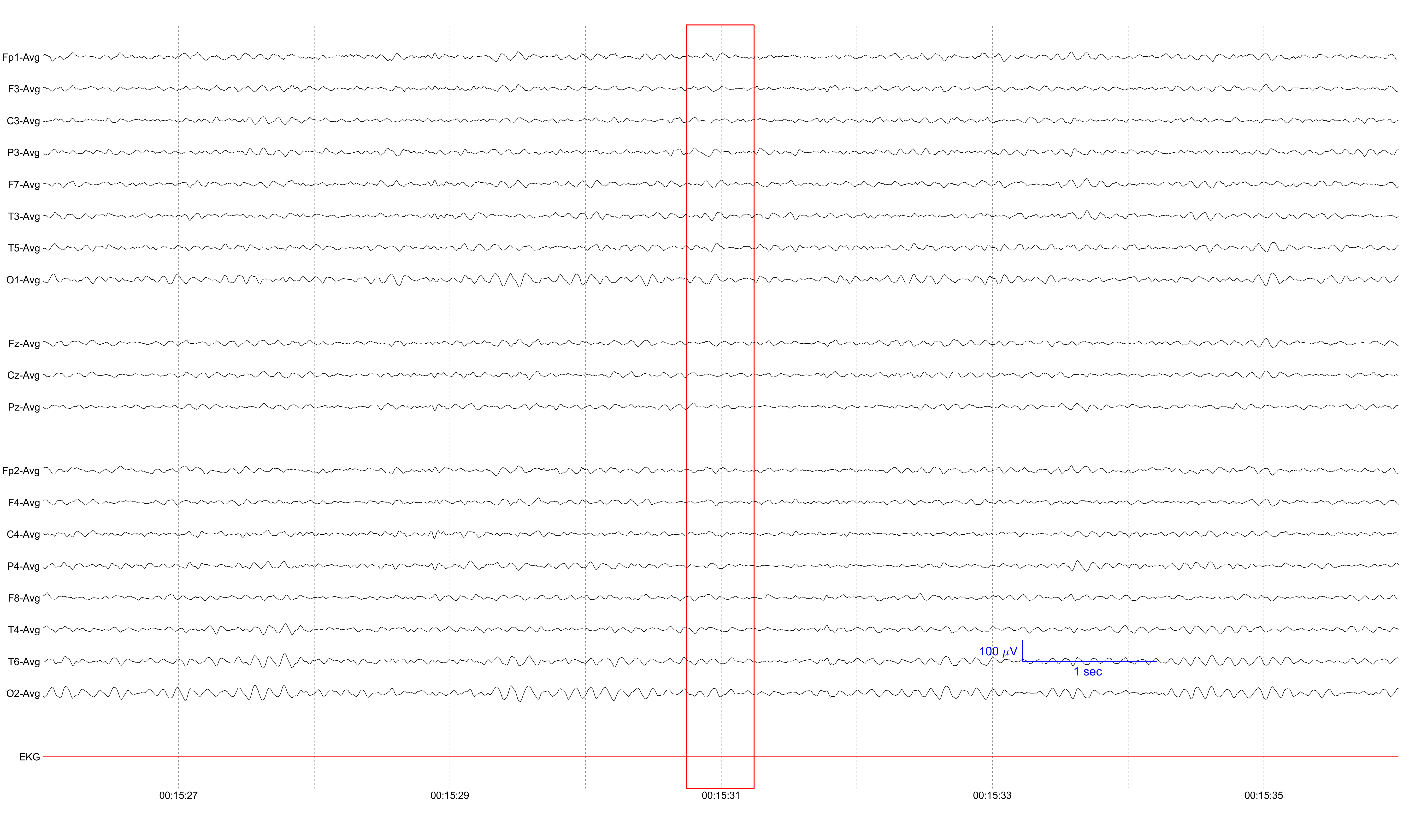

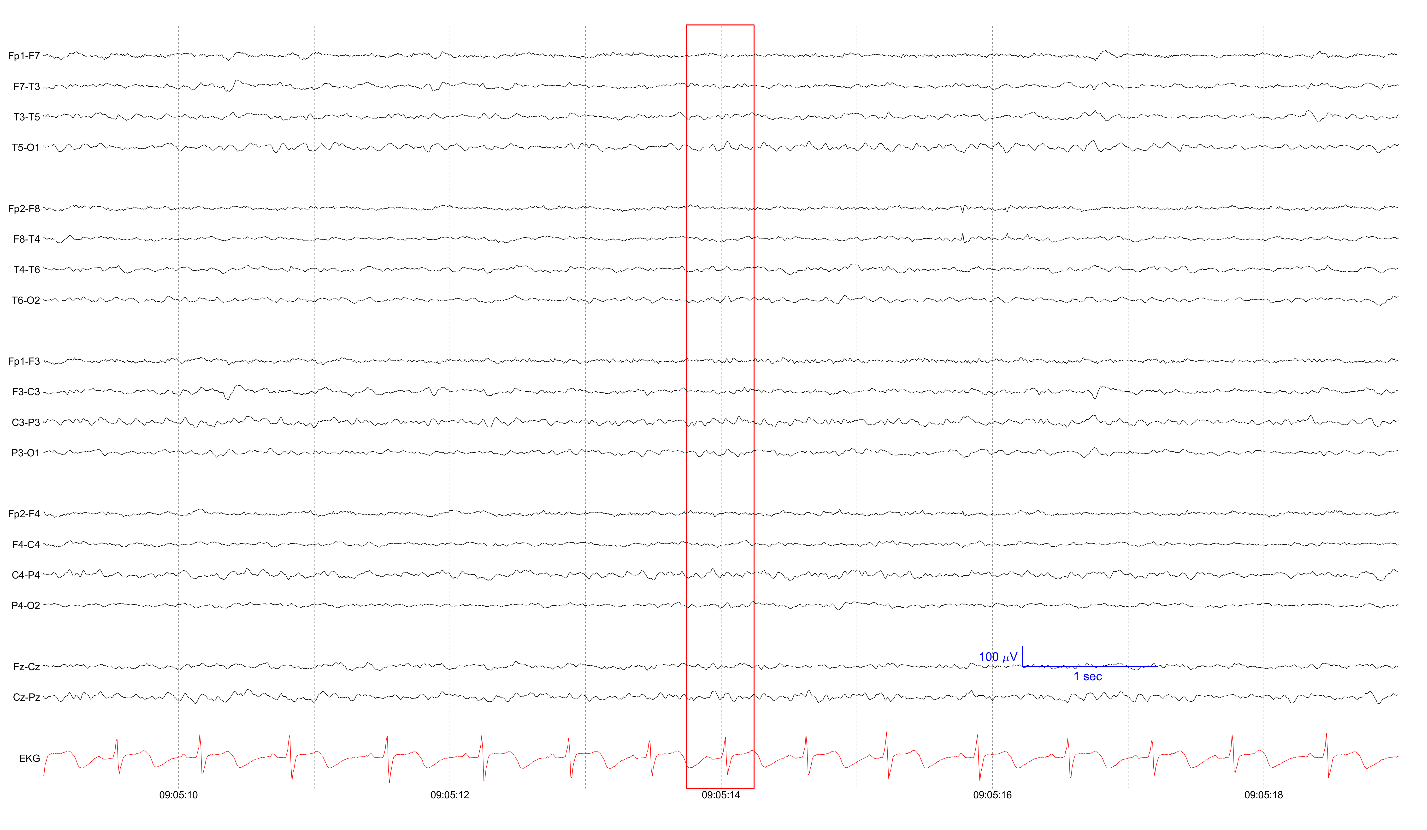

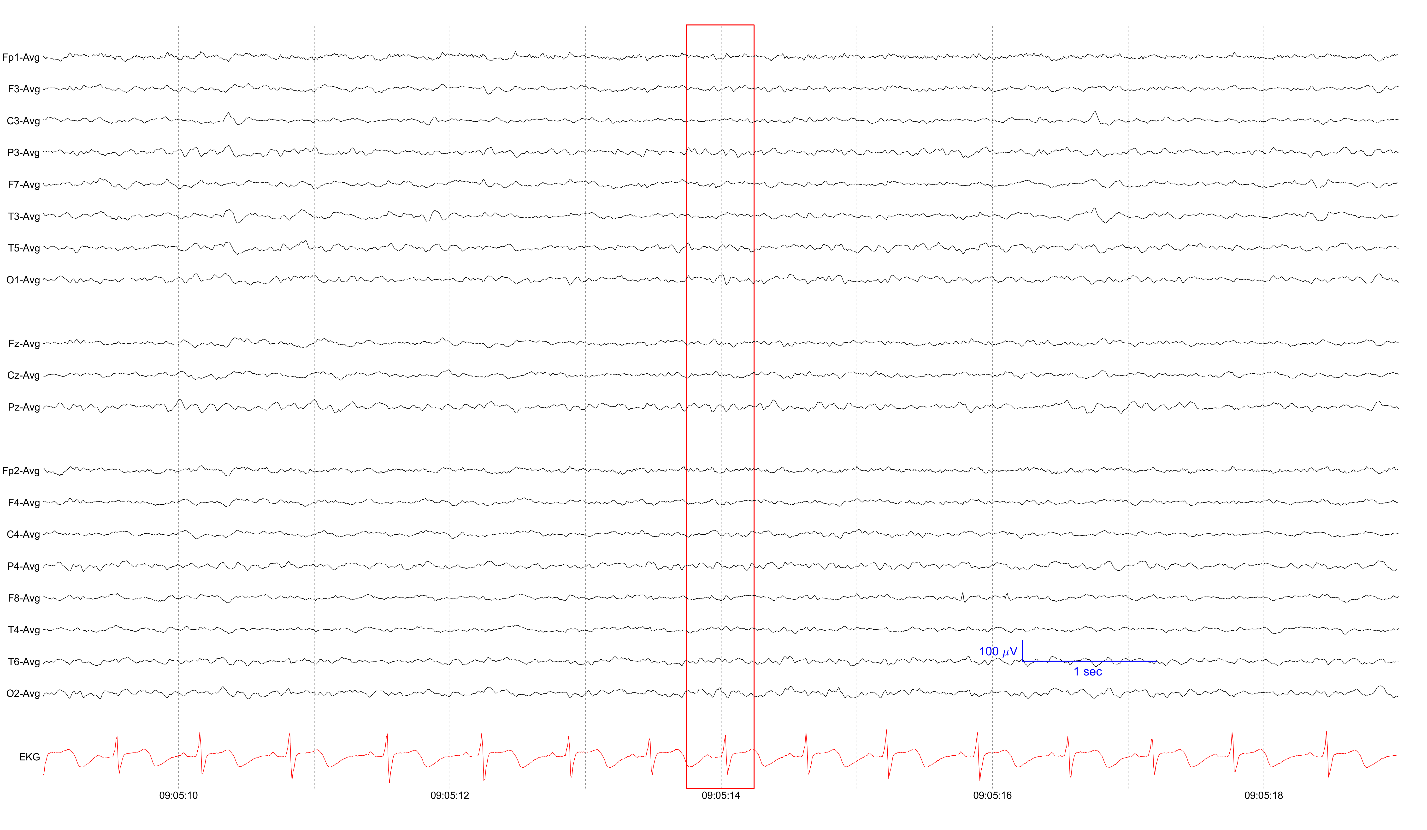

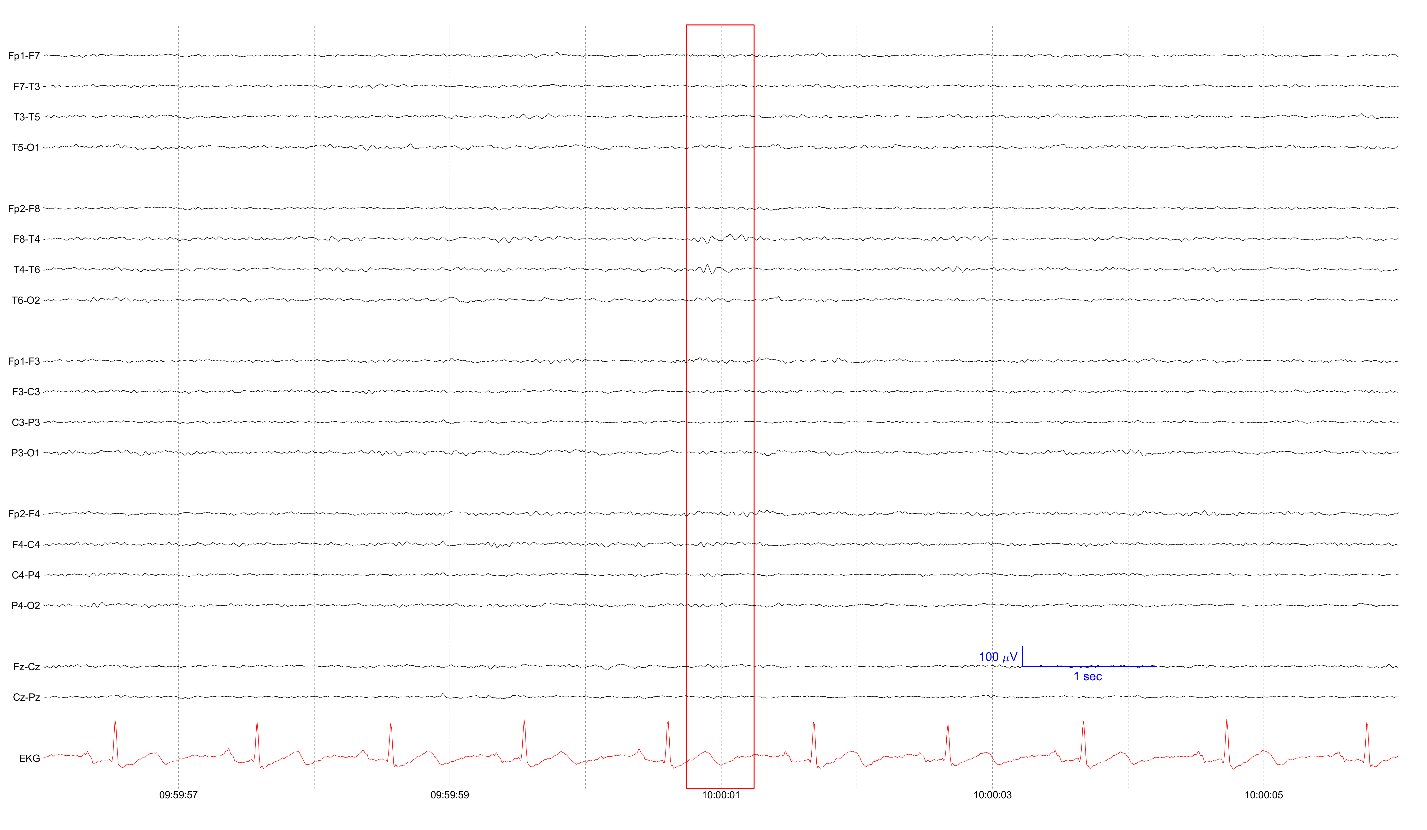

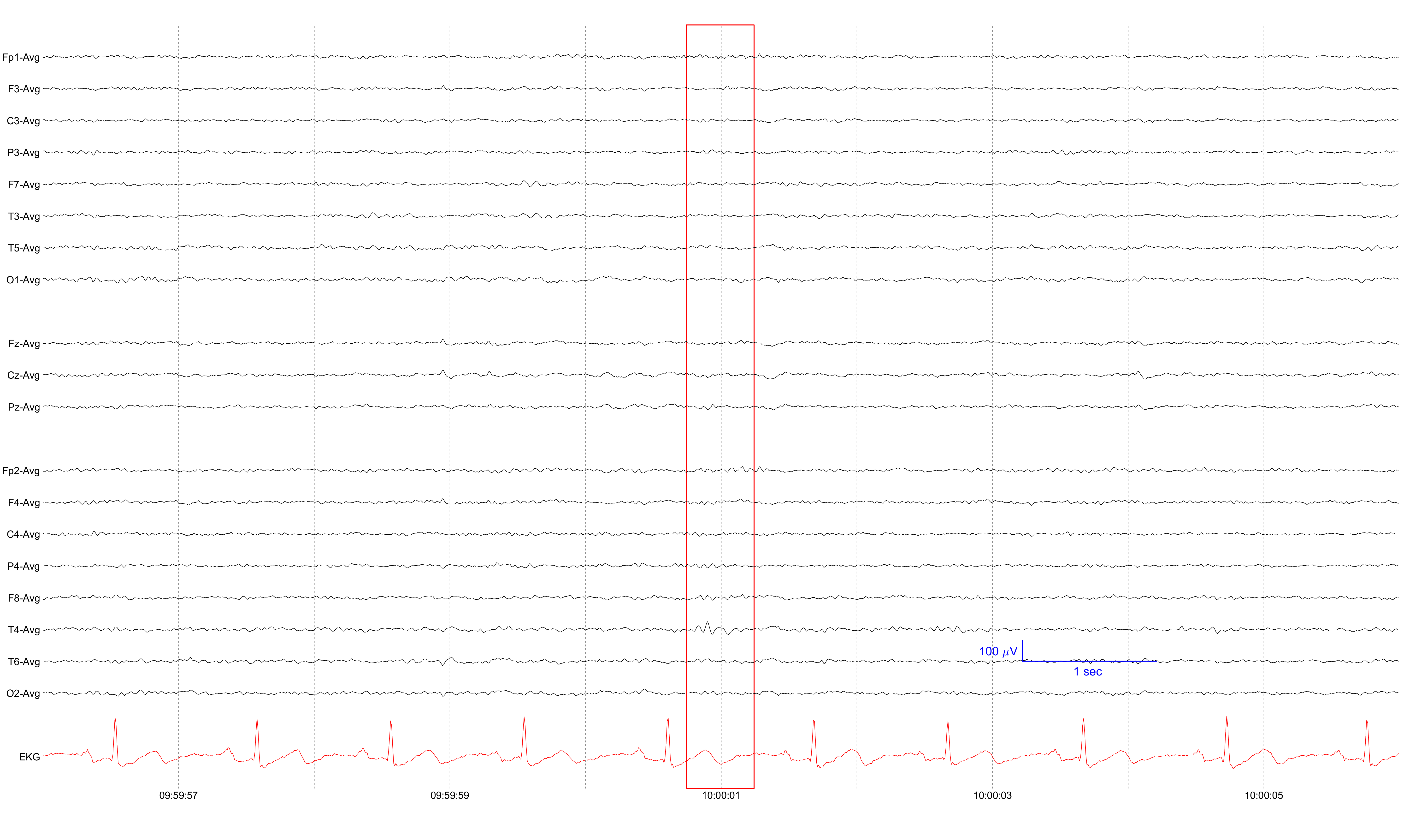

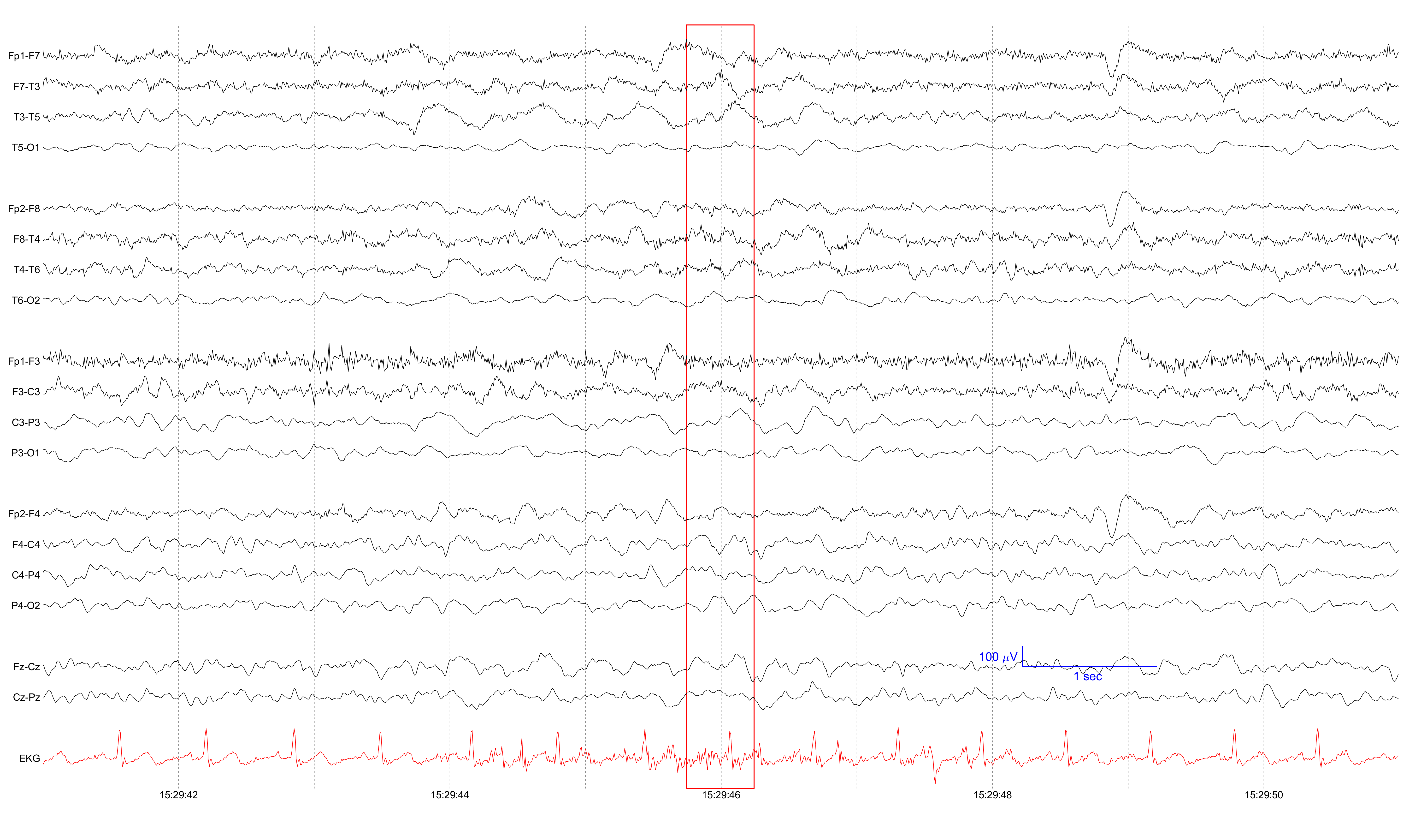

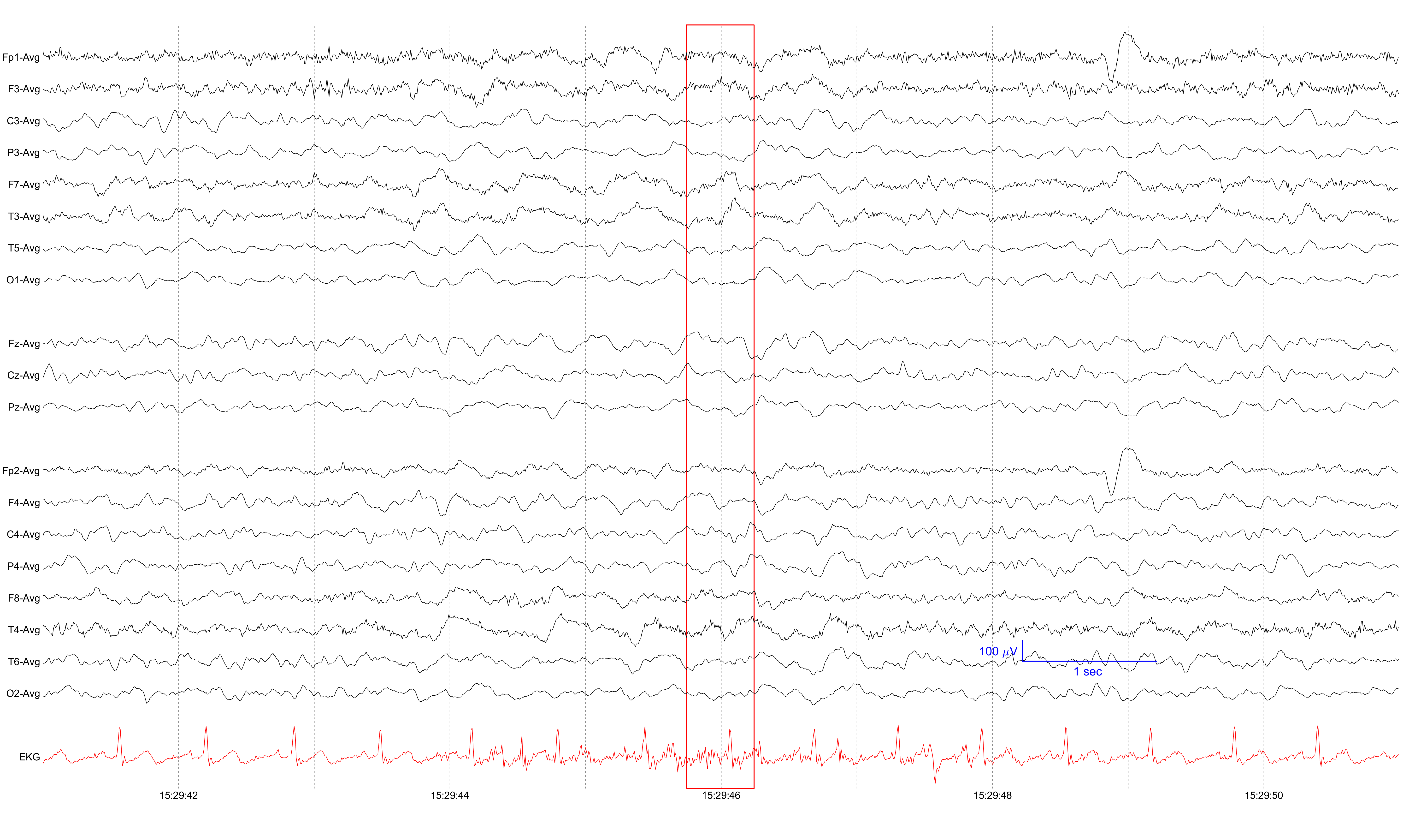

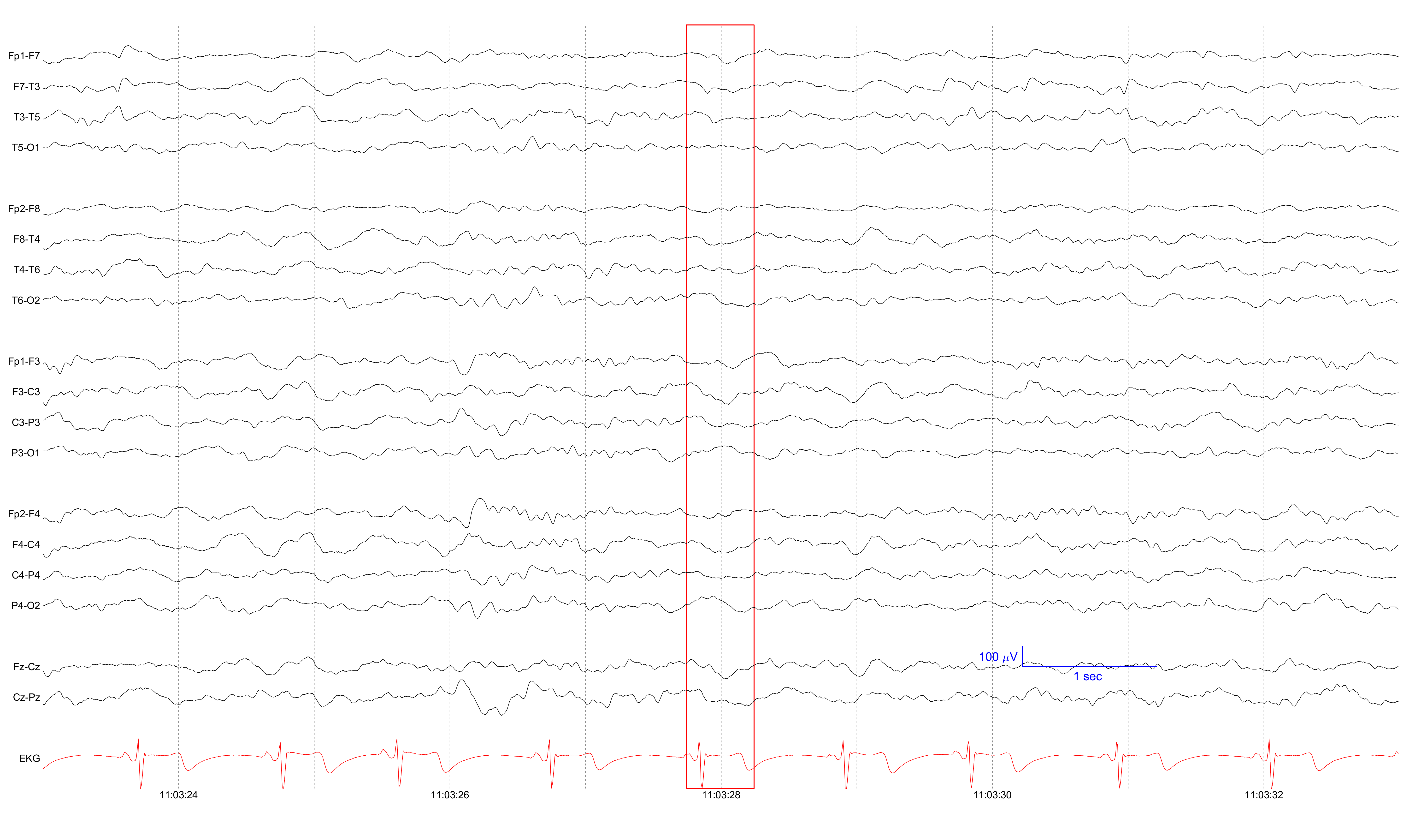

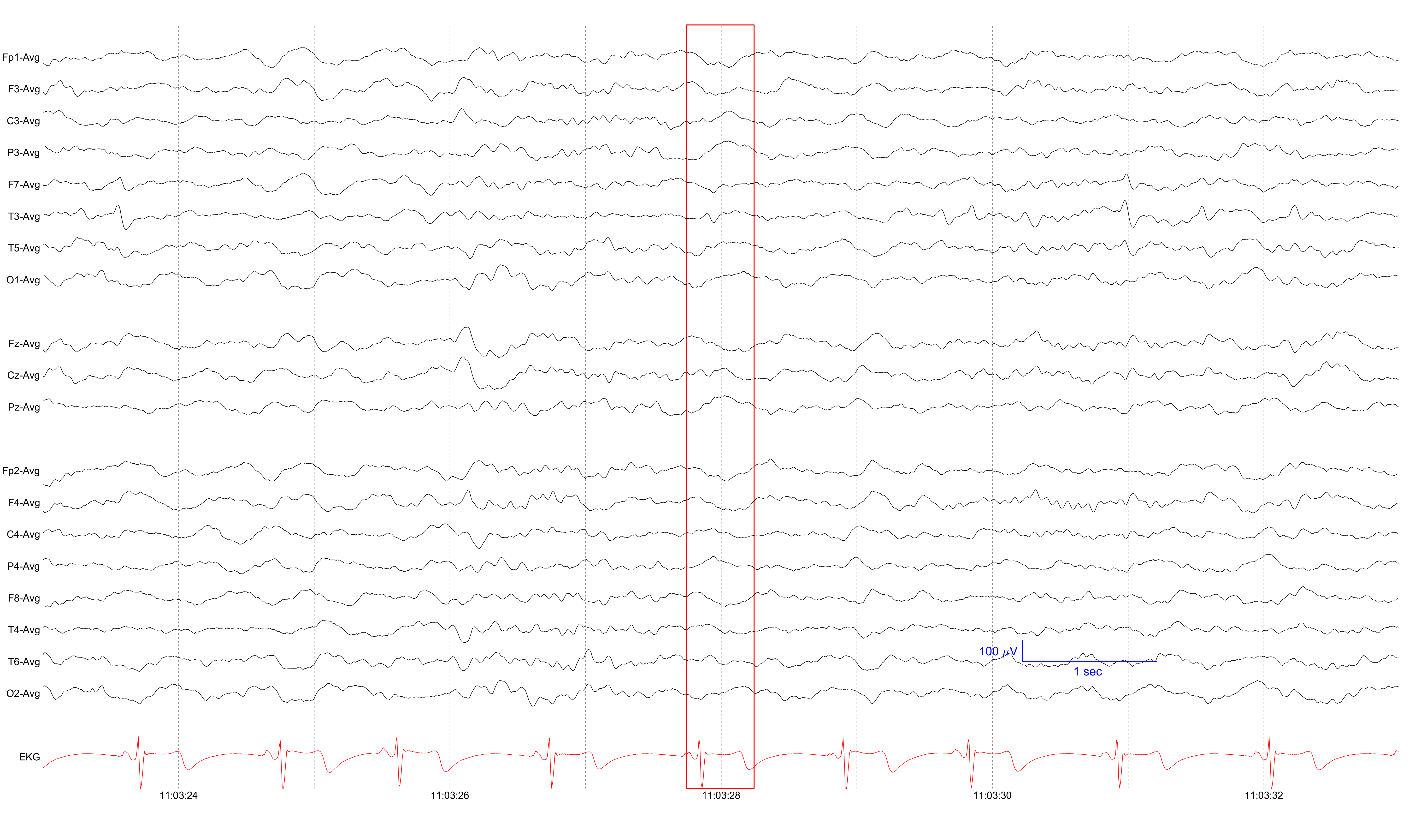

Supplement: Supplementary data 1 [file mmc1.docx]
